# Global vulnerability of marine mammals to global warming

**DOI:** 10.1038/s41598-019-57280-3

**Published:** 2020-01-17

**Authors:** Camille Albouy, Valentine Delattre, Giulia Donati, Thomas L. Frölicher, Severine Albouy-Boyer, Marta Rufino, Loïc Pellissier, David Mouillot, Fabien Leprieur

**Affiliations:** 1 IFREMER, unité Ecologie et Modèles pour l’Halieutique, rue de l’Ile d’Yeu, BP21105, 44311 Nantes, cedex 3 France; 2 MARBEC, Univ Montpellier, CNRS, Ifremer, IRD, Montpellier, France; 3 Landscape Ecology, Institute of Terrestrial Ecosystems, ETH Zürich, 8092 Zürich, Switzerland; 4 Swiss Federal Research Institute WSL, 8903 Birmensdorf, Switzerland; 5 Climate and Environmental Physics, Physics Institute, University of Bern, Bern, Switzerland; 6 Oeschger Centre for Climate Change Research, University of Bern, Bern, Switzerland; 7 Cadres en Mission Nantes, Nantes, France; 8 MARE - Marine and Environmental Sciences Centre, Faculty of Sciences, University of Lisbon, Campo Grande, 1749-016 Lisboa Portugal; 9 CCMAR, The Centre of Marine Sciences, Universidade do Algarve, Campus de Gambelas, 8005-139 Faro, Portugal; 10 Institut Universitaire de France, Paris, France

**Keywords:** Ecology, Biodiversity, Climate-change ecology

## Abstract

Although extinctions due to climate change are still uncommon, they might surpass those caused by habitat loss or overexploitation over the next few decades. Among marine megafauna, mammals fulfill key and irreplaceable ecological roles in the ocean, and the collapse of their populations may therefore have irreversible consequences for ecosystem functioning and services. Using a trait-based approach, we assessed the vulnerability of all marine mammals to global warming under high and low greenhouse gas emission scenarios for the middle and the end of the 21^st^ century. We showed that the North Pacific Ocean, the Greenland Sea and the Barents Sea host the species that are most vulnerable to global warming. Future conservation plans should therefore focus on these regions, where there are long histories of overexploitation and there are high levels of current threats to marine mammals. Among the most vulnerable marine mammals were several threatened species, such as the North Pacific right whale (*Eubalaena japonica*) and the dugong (*Dugong dugon*), that displayed unique combinations of functional traits. Beyond species loss, we showed that the potential extinctions of the marine mammals that were most vulnerable to global warming might induce a disproportionate loss of functional diversity, which may have profound impacts on the future functioning of marine ecosystems worldwide.

## Introduction

During the past few decades, the Earth has entered a new era of rapid and potentially irreversible climate warming due to the positive radiative imbalances triggered by greenhouse gas emissions from human activities^1,2^. The oceans have taken up 93% of the extra energy^1^ that has been accumulated in the Earth system in recent decades, and its temperature has increased much faster since 1991 than has been recorded previously^2^. In addition, changes in ocean temperature also affect the sea level, sea ice extent and salinity (through changes in precipitation and evaporation). All these changes were found to have negative impacts on marine biota^3,4^ and especially on marine mammals (e.g.^5–10^).

One of the most common responses of marine mammals to temperature changes is shifts in their spatial distributions, which could result in modifications of the ranges of the species (e.g.^11–13^). For example, Bryde’s whales (*Balaenoptera brydei)*, a widely spread subtropical and tropical species, was increasingly detected in the cooler waters off southern California during the period from 2000–2010^14^. In contrast, the white-beaked dolphin (*Lagenorhynchus albirostris*), a cold-water species, has reduced its range and is declining in abundance^12^. As mentioned by Elliott & Simmonds^12^, geography could constrain species with low dispersal ability to a particular area. For example, the distribution of an endemic species of porpoise, the vaquita (*Phocoena sinus*), is limited to the northern end of the Gulf of California. Changes in water temperatures could alter the life cycles of the prey of marine mammal and provoke mismatches between the abundance of prey and those of marine mammals. This situation could be particularly critical for migratory species that travel long distances between feeding and breeding areas^12^ and species that depend on prey as a source of protein for lactation or for weaned calves^12,15^. The increase in ocean temperatures could also affect the reproductive success of marine mammals, as has been reported for female sperm whales, which have lower conception rates after long exposure to higher sea surface temperatures (SSTs) than usual. Finally, increases in ocean temperatures can have direct impacts on the survival rates of marine mammals (e.g.^16,17^) by increasing the stress of organisms, fostering the development of pathogens^13^ and increasing the propagation of pathogens to new species by causing species to experience range shifts^13^. Despite the large potential impacts of ocean warming on marine mammals, the global vulnerability of marine mammals to global warming is poorly understood. This is an important knowledge gap because 37% of marine mammals are currently considered endangered by the IUCN (3 species are labeled as critically endangered, 13 as endangered and 12 as vulnerable^18,19^). In addition, marine mammals are important drivers of ecosystem functioning^13^ and fulfill key ecological roles worldwide, mostly via trophic dynamics due to their role as consumers at most trophic levels and their role in nutrient cycling (e.g.^20–24^).

Given the substantial observed and projected decline in Arctic sea ice cover^1^, it has been generally assumed that Arctic marine mammals were the most vulnerable in the face of climate change (e.g.^20,25,26^). However, marine mammals living in temperate regions could also be negatively affected as warmer sea temperatures can modify the phytoplankton blooms in spring and deregulate the whole food chain by reducing the amount of input energy in the ecosystem^20^. Identifying the most vulnerable species worldwide under projected global warming and the regions where these species are concentrated could be useful for the prioritization of species-level conservation plans (e.g.^27,28^). Furthermore, exploring the potential consequences of climate change at the assemblage level would enable the assessment of the potential losses of species diversity and allow planning of better conservation strategies. To that end, this level of investigation often requires going beyond species richness as this metric fails to consider that species may have different ecological roles and evolutionary heritages (see^29^). Thus, focusing on the functional and phylogenetic components of biodiversity should be preferred to provide an integrated approach for biodiversity conservation^30–33^. Phylogenetic diversity reflects the evolutionary history of a given assemblage and is important for explaining the role of species interactions and biogeographic histories in structuring communities^34^. Functional diversity enables the quantification of the value and range of species traits that influence the performances of certain species and thus the ecosystem functioning^35^. Hence, measuring phylogenetic and functional diversity together as complementary components of biodiversity is essential for understanding the complete structures, compositions and dynamics of natural communities^36–39^.

In this study, we assessed the vulnerability of marine mammals under future global warming using a trait-based approach^25,40,41^ (see Supplementary Methods 1 and 2). We first built a global index of the intrinsic sensitivity of all marine mammals to global warming, i.e., the magnitude of the species-specific responses to ocean warming from the inherent biological and ecological features. We then proposed an exposure index that would quantify the magnitude of ocean warming within a the geographical range of a species using the projections from 11 different global Earth system models under two future greenhouse gas emission scenarios: a low greenhouse gas emission scenario (Representative Concentration Pathway (RCP) 2.6 with a radiative forcing of 2.6 in the year 2100), and a high greenhouse gas emission scenario (RCP8.5). We used sea surface temperature (SST) as a proxy for ocean warming as this variable was found to be a good predictor of marine mammal distributions^42–44^. Finally, we derived a vulnerability index as the product of the intrinsic sensitivity and the exposure to ocean warming^33^. From this index, we also identified the global geographic hotspots of marine mammal vulnerability to global warming. Finally, we assessed the effects of the potential extinctions caused by climate change on the phylogenetic and functional diversity of marine mammals. For this, we compared several scenarios by removing species either at random or according to their vulnerability to global warming or their IUCN Red List extinction risk.

## Methods

### Intrinsic sensitivity to global warming

We evaluated the intrinsic sensitivity of marine mammal species to ocean warming using a trait-based approach^40,45–48^. The species sensitivity to ocean warming was defined as the inability of the species to persist in its habitat^40,48^. Based on an extensive review of the literature, we compiled information on fifteen traits in five main categories (feeding, habitat, reproduction, social behavior and biology) for 123 marine mammal species (see full description in Supplementary Method 1)^28^. First, we considered the traits that have been commonly used in climate change vulnerability assessments^40^ and for a wide range of taxa (see^40,45–48^, i.e., the three dimensions of rarity^49^: geographical range-related variable^50^, diet specialization and habitat specialization, which reflect the plasticity of a species^51^). Second, we accounted for specific traits that are known to mediate the sensitivity of marine mammals to climate change, such as traits related to reliance on pack ice^25^. Several traits specific to habitat requirements were obtained through analysis of the IUCN geographical range data (http://iucnredlist.org) and the Bio-ORACLE raster environmental spatial data (http://www.oracle.ugent.be; see Supplementary Method 1 for more details).

To provide a quantitative ranking of species sensitivity to global warming, we extended the methodology proposed by^25^, in which each considered trait was associated with specific quantitative ranking criteria that were evaluated on a three-point scale (with 0 being the least sensitive and 2 being the most sensitive). The ranks of the quantitative traits were evaluated by dividing the ranges of the values into three equal parts. For those quantitative traits, we performed a statistical sensitivity test on the variation of ranks from 1% to 33% (see Supplementary Method 2). When considering the qualitative traits, the modalities were ranked according to the relative influence of each modality on sensitivity to ocean warming. For example, epipelagic feeders are expected to be more sensitive to ocean warming than benthic or mesopelagic feeders since the upper layer of oceans are more exposed to warming than the deep layers^1^ (Table S1). Hence, the epipelagic modality of the ‘habitat vertical specialization’ trait was labeled as 2 (most sensitive), while the benthic and mesopelagic modalities were both labeled as 1, and the habitat-generalist species were 0 (least sensitive). The sum of all trait values (between 0 and 2) resulted in an overall species-specific sensitivity ranking that allowed direct comparison between species. The resulting values were then divided by the maximum sensitivity value to set the sensitivity index between 0 (least sensitive species) and 1 (most sensitive species).

### Exposure and vulnerability to climate change

The vulnerability of the species to global warming (V) was defined as the product of the intrinsic sensitivity (S) multiplied by the exposure (P)^40^:

$${\rm{V}}={\rm{P}}\times {\rm{S}}$$

Through this method, a species that was intrinsically sensitive to global warming would be considered vulnerable only if it was actually exposed to any change in temperature within its geographical range. Exposure was defined as the magnitude of the changes in SST within the geographical range of each species between the baseline in the present-day period (1971–2000) and the values in a near-future period (2030–2059) and a period at the end of 21^st^ century (2070–2099)^52^. To assess the uncertainties in the models and scenarios, we used 11 different Earth system models that participated in the Coupled Model Intercomparison Project phase 5 (CMIP5^53^) and that were run for the 1861–2100 period under the historical, RCP8.5 and RCP2.6 scenarios^54^. We regridded the model output data onto a regular 1° × 1° grid (∼10,000 km²) by using the *Krige* function of the R package gstat. We selected the following CMIP5 ESMs: MRI-CGCM3, IPSL-CM5A-LR, GFDL-ESM2G, GFDL-ESM2M, IPSL-CM5A-MR, MIROC-ESM, MPI-ESM-LR, GFDL-CM3, CSIRO, and CanESM2. We selected high and low greenhouse gas emission scenarios among the set of RCPs that were run with the CMIP5 models to explore the impacts of different socioeconomic climate policies on the climatic projections^55^. RCP8.5 is a high-carbon emission, business-as-usual scenario with a net radiative forcing of 8.5 W/m^2^ by 2100 and a simulated global atmospheric surface temperature change of approximately 3.7 ± 0.7 °C relative to the temperature from 1986–2005. RCP2.6 is a strong emission mitigation scenario with a global mean net radiative forcing of 2.6 W/m^2^ by 2100 and a simulated global atmospheric surface temperature change of approximately 1.0 ± 0.4 °C^56^ relative to 1986–2005. The Earth system model output was regridded onto a regular 1° × 1° grid (∼10,000 km²) using the *Krige* function of the R package gstat, and we extracted the average value of the SST for both the baseline records and the projected SST from each model and for the two emissions scenarios.

We then calculated the changes in the SST at the global scale within the geographical range of each species from the baseline to the two future periods for both the RCP2.6 and RCP8.5 scenarios. We downloaded the geographical range maps from the IUCN (http://www.iucnredlist.org) for the 123 studied species and then derived a presence/absence matrix by overlapping the geographical ranges and counting how many species occurred in each grid cell (1° × 1° grid cells). To quantify the degree of exposure to ocean warming at the species level, the distributions of the absolute values of *δ* SST were divided into three equal parts according to statistical considerations. The lowest category was coded as 0, the middle category as 1 and the upper as 2. For each species geographical range that was divided into grid cells (1° × 1°), we counted the relative number of cells occurring in each category and deducted the exposure value according to the following equation:

$${\rm{P}}={\rm{a}}\times 0+{\rm{b}}\times 1+{\rm{c}}\times 2$$

where *a*, *b* and *c* are the percentage of cells in the lower, middle and upper categories, respectively. This index accounts for the heterogeneity in *δ* SST throughout the geographical range of a species and is independent of the geographical range size. Consequently, a species that will face great temperature changes (either increases or decreases) in a large proportion of its range was categorized as highly exposed to climate change, while a species that will likely face lower *δ* SST within its whole range was categorized as less exposed.

### Diversity erosion scenarios

We investigated the potential erosion of marine mammal diversity under global warming through two complementary biodiversity components: the phylogenetic and functional diversities. Phylogenetic diversity was measured with the PD index^57^ as the minimum total length of all the phylogenetic branches required to represent a given set of taxa on the phylogenetic tree^57^. The PD calculation was applied to a pruned supertree of mammals^58^. However, this supertree contained a large number of polytomies, which implied potentially poor branch length estimates in parts of the tree. To account for this phylogenetic uncertainty in our analyses, we also quantified PD using 100 trees that were chosen at random from the Bayesian posterior distribution of the fully resolved trees that were generated by a polytomy resolver^59^.

The functional diversity was quantified using an index of functional richness (FRic^60^). The FRic index relies on a multidimensional Euclidean space where the axes are functional traits (or factorial axes from a principal coordinates analysis (PCoA) computed using these traits) along which species are placed according to their trait values^60^. This index measures the volume of the functional space that is occupied by a given species assemblage by calculating the convex hull volume, which is defined by the species at the vertices of the functional space that encompasses the entire trait space filled by all species in the assemblage^60^. To calculate the FRic index, we used 13 functional traits, i.e., main diet, foraging depth range, foraging location, fasting strategy, female sexual maturity, weaning, gestation length, interval between litters, breeding sites, social group size, social behavior, adult maximum body mass and sexual dimorphism (see^28^ for full details). These traits covered five functions of marine mammals (i.e., feeding, reproduction, habitat, social behavior and intrinsic biology) and reflected the trade-offs in resource allocation^20^. The pairwise functional distances between the species were computed using Gower’s distance, which allows the mixing of different types of variables while weighting the functional traits in order to give equal weight to each function (i.e., reproduction, social behavior, biology, feeding and habitat). Then, a principal coordinates analysis (PCoA) was performed using this functional distance matrix^61^. According to the metric proposed by^62^ to evaluate the quality of a functional space (i.e., the extent to which it is a faithful representation of the initial functional trait values), we retained the first four principal axes of the PCoA to build the multidimensional functional space. We preferred to use the FRic index instead of the FD index, which is based on a functional dendrogram, as a recent study showed that the FD index may lead to biased assessments of functional diversity and inaccurate ecological conclusions^62^. In addition, additional analyses showed that the distances derived from a UPGMA dendrogram did not faithfully represent the initial Gower’s distances.

For each diversity index (PD and FRic), the diversity was initially measured for the global pool of marine mammals. Extinctions were simulated by removing individual species from the global pool and then recalculating the diversity of the remaining pool. The order of the species to be removed from the global pool varied among four types of scenario. First, we developed a vulnerability scenario in which the order of the simulated extinctions was given by the ranking of the vulnerability of a species to climate change, where the most vulnerable species were assumed to go extinct first. Second, we constructed 999 random scenarios in which the order of the species to be removed from the pool was chosen randomly. We computed the mean and SD values of these scenarios. Third, we implemented a scenario that coupled the IUCN classification and the ranking of vulnerability to climate change, and we simulated extinctions by ordering the groups of species according to the IUCN categories (i.e., critically endangered (CR), endangered (EN), vulnerable (VU), near threatened (NT), least concern (LC) and data deficient (DD)). Within each group of species in the same IUCN category, the species were ranked according to their vulnerabilities to climate change. Hence, the species that was simulated to go extinct first was the most vulnerable to climate change among those classified as critically endangered. Finally, we implemented 999 random IUCN scenarios where extinction was predicted by ordering the groups of species according to the IUCN categories. We randomized the order of species removal within each group. We used the mean and SD for the graphical representation.

### Functional and phylogenetic originality

To explore whether the most vulnerable species to climate change were the most “original” from a functional or phylogenetic point of view, we calculated two distinct metrics. First, we quantified the phylogenetic originality (or evolutionary distinctiveness) of each species using a metric that represented the relative contributions of the species to the phylogenetic diversity^63^. We calculated the mean evolutionary distinctiveness per species using the 100 resolved phylogenetic trees. Second, we derived the functional originality of each species from Gower’s distance matrix^36^. This metric was calculated as the distance between each species and its nearest neighbor in the functional space and hence represented the degree of the isolation of a species in the functional space occupied by a given assemblage^36^.

## Results

### Projected climate change

The CMIP5 Earth system models project an increase in global SST of 0.7 ± 0.51 °C (RCP2.6) and 2 ± 1.3 °C (RCP8.5), on average, between the baseline period (1971–2000) and the end of the 21^st^ century (2070–2099). However, the projected increase in SST will not be homogeneous throughout the world ocean (see Figs. S1–S3 for the period 2030–2059). For example, by the end of the century and according to the RCP85 scenario, the Barents Sea, the Bering Sea and the Tasmanian Sea are projected to experience increases in SST of up to 4.5 °C, while other regions such as the Labrador Sea or the Southern Ocean are projected to show low levels of warming or even decreasing SSTs (Figs. S1, S2).

### Sensitivity, exposure and vulnerability to climate change

The index of sensitivity to ocean warming, which ranged ranging from 0 to 1, was based on fifteen species traits reflecting the biological and demographic responses of marine mammals to climate change (see Methods and Supplementary Method 1). The sensitivity of the species to ocean warming (S) ranged from 0.35 to 1 (see Fig. S4; Table S2), with a mean value of 0.69 (sd = 0.12). The walrus (*Odobenus rosmarus*) and the North Pacific right whale (*Eubalaena japonica*) were the most sensitive species (S = 1), followed by the gray whale (*Eschrichtius robustus;* S = 0.92), the crabeater seal (*Lobodon Carcinophaga;* S = 0.92), the North Atlantic right whale (*Eubalena glacialis*, S = 0.88), the dugong (*Dugong dugon*, S = 0.88) and the narwhal (*Monodon Monoceros;* S = 0.88; Table S2).

At the end of the 21^st^ century, according to the RCP2.6 and RCP8.5 scenarios, respectively, the exposure of the species to climate change ranged from 0 (not exposed to SST variation throughout the geographic range of the species between the baseline period and the end of the century) to 0.92 and 1.2 (most exposed to ocean warming), with mean values of 0.34 and 0.62, respectively (see Fig. S4; Tables S2, S3).

**Figure 1:**
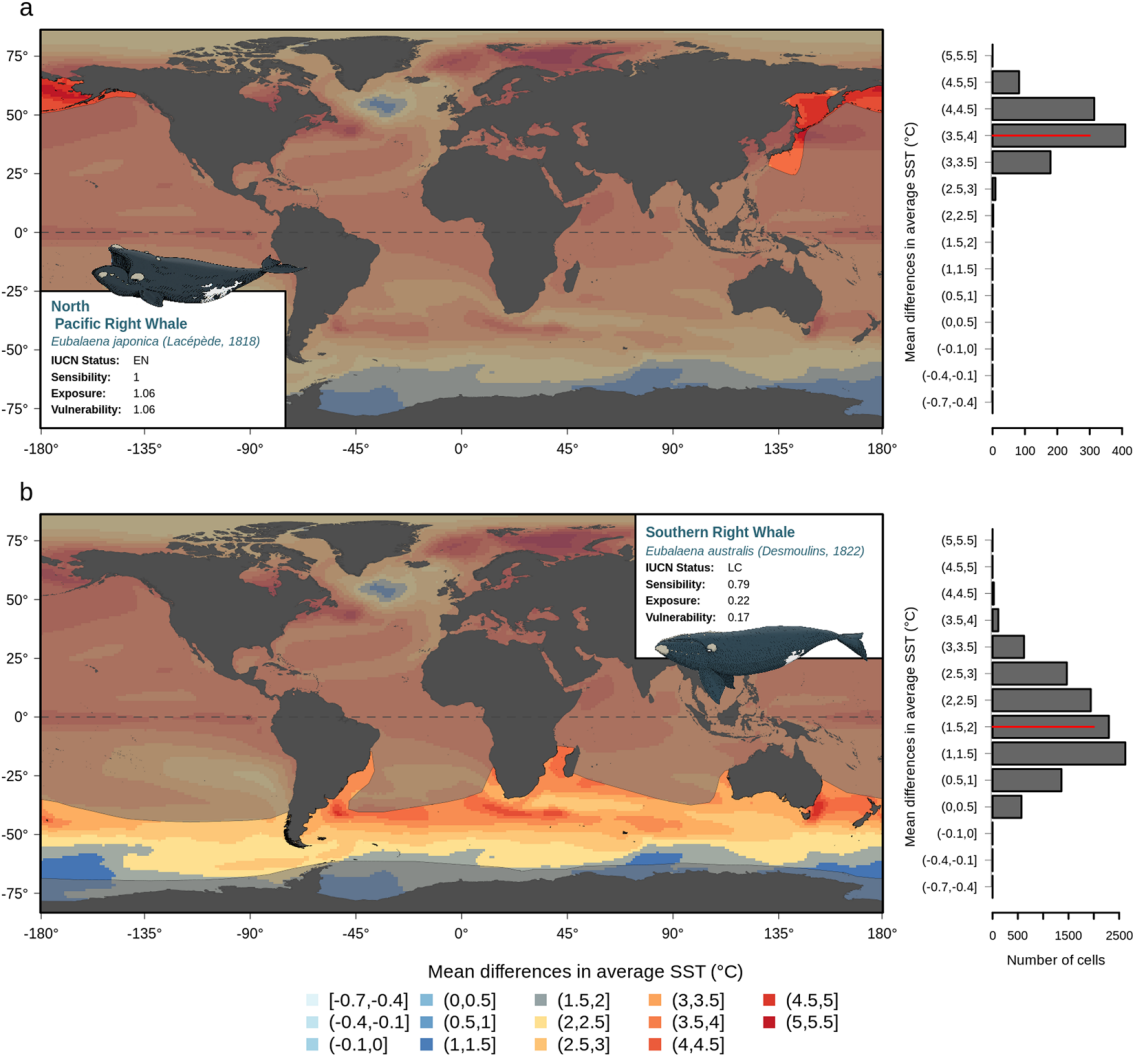
Projected climate change within the geographical species ranges. Projected climate change within the geographical range of the North Pacific right whale (**a**) and the Southern right whale. (**b**) Colors show the magnitudes of the difference in the mean sea surface temperature between the baseline period (1971–2000) and the end of the century (2070–2099) according to the RCP8.5 scenario. The geographical range is represented in bright colors that contrast with the dark background. These maps and barplots allow visualization of the exposure, i.e., the degree of climate change (either warming for positive values of SST or cooling for negative values) that a species is likely to face within its range. Maps were created using R 3.6.0 software (https://www.r-project.org/).

Combining the sensitivity and exposure indices allowed us to assess the vulnerability of marine mammals to global warming. This was best exemplified by the whale species belonging to the Eubalaena genus. For example, the North Pacific right whale (*Eubalaena japonica*) and the southern right whale (*Eubalaena australis*) were both highly sensitive to climate change according to their intrinsic biological and ecological characteristics but showed contrasting exposure to climate change within their current geographical ranges (Fig. 1). In fact, the North Pacific right whale would be more exposed to climate change at the end of the 21^st^ century than the southern right whale according to both RCP scenarios, which makes the former species more vulnerable to global warming (Fig. 1). Overall, at the end of the century, the vulnerability index ranged from 0 to 0.74 and 1.06, according to the RCP2.6 and RCP8.5 scenarios, respectively, with mean values of 0.14 (±0.12 sd) and 0.42 (±0.22 sd) (see Fig. S4; Tables S2, S3).

**Figure 2:**
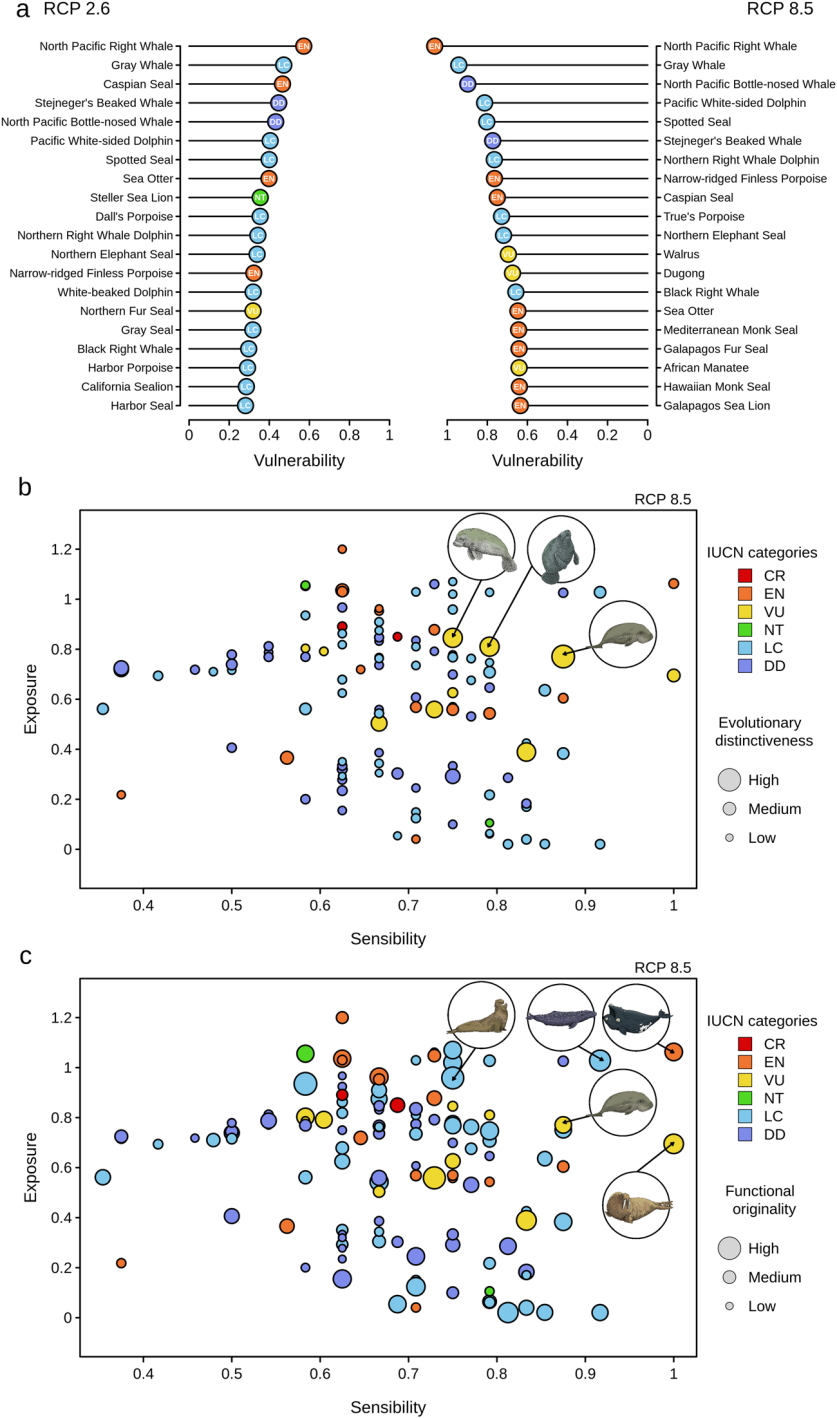
Vulnerability of marine mammals to climate change. (**a**) Ranking of the 20 most vulnerable marine mammals to climate change according to two RCP scenarios: RCP2.6 (left) and RCP8.5 (right). The current IUCN status is indicated by a color range from red (critically endangered) to light blue (least concern) and the data deficient status is shown in violet. (**b**) Relationship between the intrinsic sensibility of each marine mammal to climate change and its exposure to projected climate change within its geographical range according to the RCP8.5 scenario. Each bubble is proportional to the level of functional originality (i.e., the measure of the extent to which a given species is functionally unique among the global marine mammal fauna). In the top right corner, the drawings of the most vulnerable species also display the highest levels of evolutionary distinctiveness, namely, the dugong, the African manatee and the Florida manatee. (**c**) Relationship between the intrinsic sensitivity of each marine mammal to climate change and its exposure to projected climate change within its geographical range according to the RCP8.5 scenario. Each bubble is proportional to the level of evolutionary distinctiveness (i.e., which represents the relative contribution of a species to the evolutionary history of the global marine mammal fauna). In the top right corner, the drawings of the most vulnerable species also display the species with highest levels of functional originality, namely, the North Pacific right whale, the gray whale, the dugong, the Northern elephant seal and the walrus.

At the end of the 21^st^ century, the ranking of the 20 most vulnerable species based on the RCP8.5 scenario reveals the overrepresentation of IUCN-recognized endangered or vulnerable species (endangered = 40%, vulnerable = 15%) compared to the RCP2.6 scenario (RCP2.6: endangered = 5%, vulnerable = 5%; Fig. 2a). Despite these differences, the North Pacific right whale (*Eubalaena japonica*) and the gray whale (*Eschrichtius robustus*) were found to be the most vulnerable species to ocean warming according to both RCP scenarios (Fig. 2a). The vulnerability of a species to ocean warming had no phylogenetic signal (*Pagel λ* <0.1 regardless of the RCP scenario and the time period considered; values obtained from over 100 resolved phylogenetic trees), indicating that future climatic impacts were not clustered within particular lineages of marine mammals.

At the end of the 21^st^ century and for both RCP scenarios, no clear relationship was found between the vulnerabilities of different species to ocean warming and their levels of evolutionary distinctiveness, which represented their relative contributions to the evolutionary history (or phylogenetic diversity) in the global marine mammal fauna (Spearman rank correlation test, RCP2.6: *ρ* = 0.045, p = 0.63; RCP8.5: *ρ* = 0.025, p = 0.78; see Figs. 2b and S5). Nonetheless, a few species were simultaneously highly vulnerable to climate change and evolutionarily distinct (Fig. 2b), namely, the dugong (*Dugong dugon*), the African manatee (*Trichechus senegalensis*) and the Florida manatee (*Trichechus manatus*). Moreover, these three species are classified as vulnerable according to the IUCN Red List.

Similarly, no clear relationship was found between the vulnerabilities of different species to ocean warming and their levels of functional originality (i.e., the degree of uniqueness of the species traits in the global pool of marine mammals) for both RCP scenarios at the end of the 21^st^ century (Spearman rank correlation test, RCP2.6: *ρ* = 0.17, p = 0.07; RCP8.5: *ρ* = 0.011, p = 0.21; Fig. S5). However, contrary to the results for evolutionary distinctiveness, 9 and 17 species were both vulnerable to climate change and functionally distinct according to the RCP2.6 and RCP8.5 scenarios, respectively, at the end of the 21^st^ century. In the RCP8.5 scenario, 3 of the 17 species were classified as endangered, 10 as least concern, 1 as near threatened and 3 as vulnerable. For example, the North Pacific right whale (*Eubalaena japonica*), the gray whale (*Eschrichtius robustus*), the dugong (*Dugong dugon*), the northern elephant seal (*Mirounga angustirostris*) and the walrus (*Odobenus rosmarus*) were both vulnerable to climate change and functionally unique (Figs. 2c and S5). Two of these species were considered to be endangered or vulnerable according to the IUCN Red List, namely, the North Pacific right whale and the dugong, respectively.

**Figure 3:**
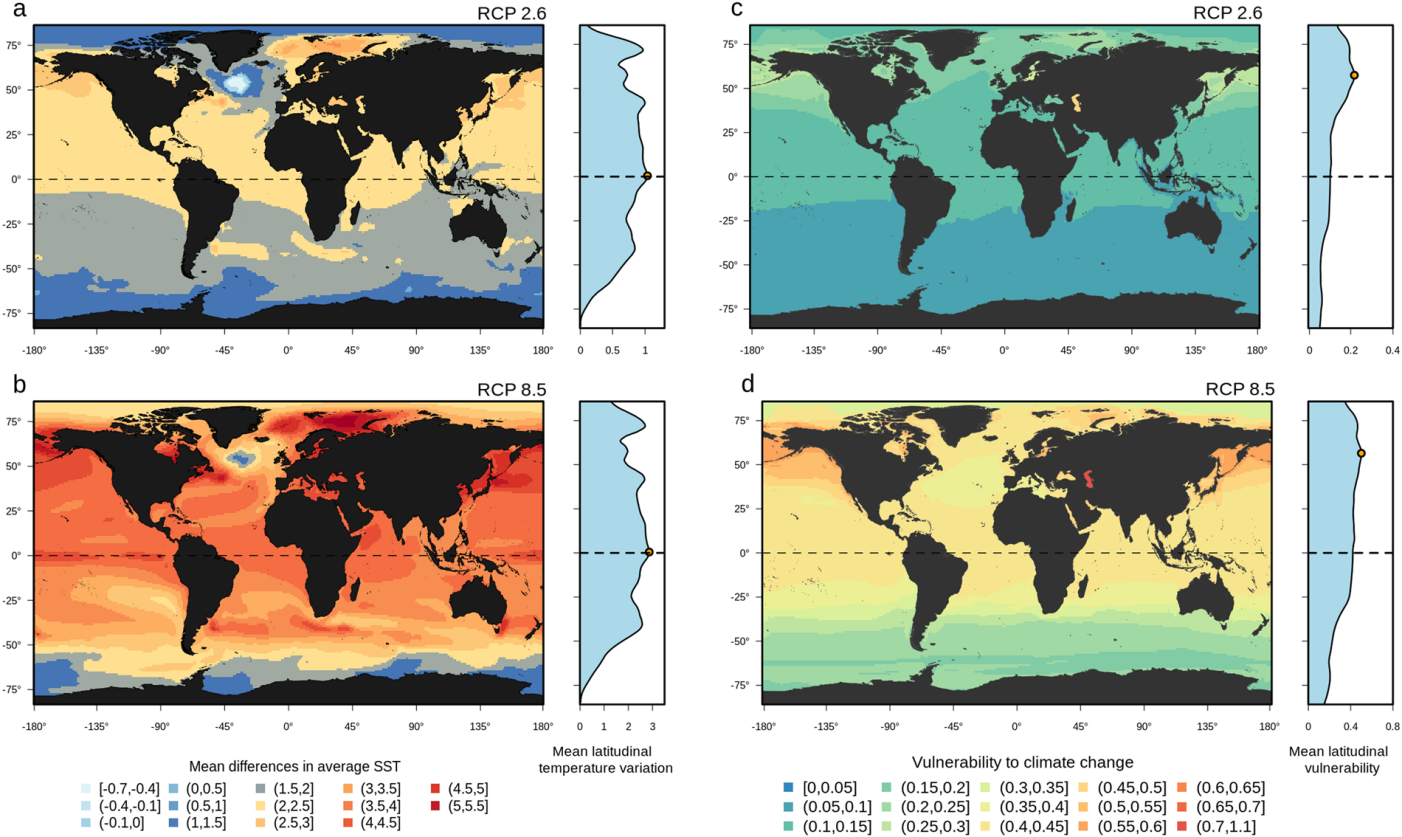
Projected changes in sea surface temperatures and the associated vulnerabilities of marine mammals. Projected changes in the sea surface temperature between the baseline period (1971–2000) and the end of the century (2070–2099) following the RCP2.6 (**a**) and RCP8.5 (**b**) scenarios as simulated by 11 different CMIP5 Earth system models (MRI-CGCM3, IPSL-CM5A-LR, GFDL-ESM2G, GFDL-ESM2M, IPSL-CM5A-MR, MIROC-ESM, MPI-ESM-LR, GFDL-CM3, CSIRO, CanESM2). Local assemblage-level vulnerability of marine mammals to climate change for the period (2070–2099) based on (**c**) the RCP26 scenario and (**d**) the RCP8.5 scenario. To evaluate the vulnerability of marine mammals to climate change at the assemblage level, we averaged the vulnerability of each species in each grid cell (1° × 1° grid ells, ∼10,000 km²). Maps were created using R 3.6.0 software (https://www.r-project.org/).

When considering the vulnerability of marine mammals to global warming at the assemblage level, our results showed a latitudinal gradient for both RCP scenarios at the end of the 21^st^ century (Fig. 3c,d). The mean vulnerability to climate change was found to be higher in the northern hemisphere, peaking at the 57°5′ and 56°5′ latitudes for RCP2.6 and RCP8.5, respectively. The areas with the highest mean vulnerabilities to climate change were the Baltic, Black and Caspian Seas; the Bering Sea; the area along the Japanese coast; and the northern part of the Pacific Ocean along the Canadian and American coasts (Figs. 3c,d, and S3 for the period from 2030–2059).

### Scenarios of functional and phylogenetic diversity erosion

**Figure 4:**
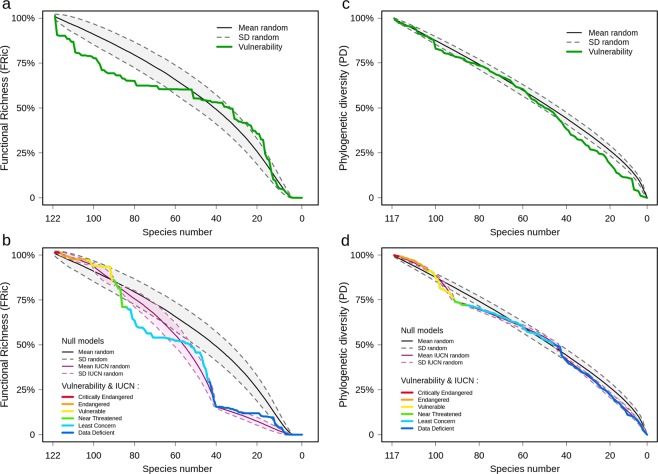
Scenario of the potential loss of functional richness and phylogenetic diversity in the global marine mammal fauna. Potential loss of functional richness (**a**,**b**) and phylogenetic diversity (**c**,**d**) in the global marine mammal fauna according to the RCP8.5 scenario for the end of the century (2070–2099). The upper graphs (**a**,**b**) show two erosion scenarios; one was repeated 999 times with a randomly selected order of species extinctions (dark continuous line) and is shown with the corresponding standard deviation, while the second had the species extinctions occur in order of their vulnerabilities to climate change (green continuous line). The lower graphs (**c**,**d**) show two random erosion scenarios. One was repeated 999 times with a randomly selected order of species extinctions (dark continuous line) and a second where the species were grouped according to their IUCN categories (critically endangered (CR), endangered (EN), vulnerable (VU), near threatened (NT), least concern (LC) and data deficient (DD)) prior to randomization. Finally, the lower graph presents a scenario (“Vulnerability & IUCN”, multicolor line) with the species grouped by IUCN categories and placed in decreasing order within each group according to their vulnerabilities to climate change. The ranking of the species differs between each pair of vulnerability scenarios, preventing direct comparison.

Regardless of the considered RCP, the vulnerability scenarios that consists of removing species from the global pool of marine mammals according to their level of vulnerability to climate change (i.e., from the most to the least vulnerable species), showed a faster decreases in functional diversity (i.e., the amount of functional trait space occupied by the species, as measured by the FRic index^50^) than would be expected by chance by the end of the 21^st^ century (Figs. 4a,b and S6 for the period 2030–2059). For example, the extinction of the top 20 most vulnerable marine mammals as a result of climate change, according to the RCP8.5 scenario, would induce a functional diversity loss of approximately 20% (Fig. 4a) and a decrease of 13% compared with a random loss.

The vulnerability-IUCN scenario, which consisted of removing species according to their vulnerabilities to climate change by considering the IUCN status, showed that the extinctions of species within the least concern category would induce a faster loss of functional diversity than expected by a random scenario of species removal within each IUCN category (Figs. 4b and S6). Finally, the removal of the most vulnerable species, based on both RCP scenarios for the end of the 21^st^ century, showed that the loss of phylogenetic diversity would not be different than expected with random extinctions (Figs. 4c and S6). Similar results were found when considering the IUCN-vulnerability scenario (Figs. 4d and S6).

## Discussion

Marine mammals are threatened both directly and indirectly by different human activities, such as fishing, whale watching, vessel collisions, acoustic disturbance, pollution, and modification or loss of available habitats^64^. Consequently, 37% of marine mammals are already included in the IUCN Red List (3 species are labeled as critically endangered, 13 as endangered and 12 as vulnerable^65^). However, the marine mammal vulnerability to global warming has never been assessed at the species and global levels. Here, using a species-level trait-based approach, we showed that many marine mammal species distributed across the northern hemisphere and belonging to different taxonomic groups (e.g., whales, dolphins and seals) were highly vulnerable to global warming (Fig. 3), even under a strong mitigation scenario (i.e., RCP2.6), which, according to the CMIP5 simulations, gives a two in three chance of limiting global warming to below 2 °C. A key finding from our study was that the North Pacific, which has already been identified as a hotspot of human threats for marine mammals^19,64^, is also a hotpot of vulnerability to global warming for this group. This implies that marine mammals in this region face double jeopardy from both human activities (e.g., marine traffic, pollution and offshore oil and gas development) and global warming, with potential additive or synergetic effects and as a result, these ecosystems face irreversible consequences for marine ecosystem functioning.

The marine mammals that are the most sensitive to climate change generally show marked feeding and habitat specialization, as well as reduced or fragmented geographical ranges (e.g., the dugong *Dugong dugon* and the walrus *Odobenus rosmarus*), which is consistent with the results of earlier studies that focused on diverse terrestrial and marine organisms^25,40,66–68^. Dependence on sea ice also seems to be a common denominator for many of the most sensitive marine mammals that are most sensitive to climate change^25^ (e.g., the polar bear *Ursus maritimus* and the beluga whale *Delphinapterus leucas*).

By definition, the vulnerability index was higher for species that were both sensitive and exposed to global warming. The most vulnerable species according to both future RCP scenarios was the North Pacific right whale (*Eubalaena japonica*). This species has already been identified by the IUCN as an endangered species because of the absence of evidence of a recovering trend and the extremely low estimated number of individuals (~400 in the Okhotsk Sea and ~100 in the rest of the North Pacific)^69^. While historically common in many areas of the North Pacific and Bering Sea, the North Pacific right whale was strongly depleted by intensive whaling in the mid-19^th^ century^70^. The second most vulnerable species was the gray whale (*Eschrichtius robustus*), which is classified as least concern by the IUCN, as its population size has been estimated to be above the threshold for the threatened category and the eastern subpopulation has increased over the last three generations^71^. However, the gray whale subpopulations in the western North Pacific are listed as critically endangered by the IUCN. Indeed, decades of commercial whaling led to the complete collapse of this subpopulation in much of its historical range. The Pacific right whale and the gray whale were also found to be functionally unique, i.e., with unique combinations of functional traits. Their potential extinction could have large consequences on marine ecosystem functioning^72^. For example, the gray whale can resuspend large amounts of sediment and nutrients in the water column, which in turn enhances nutrient cycling and brings some benthic crustaceans that serve as food for seabirds to the ocean surface^73^. For this species, a recent study showed that dispersal occurred between the eastern North Pacific and the North Atlantic during the Pleistocene and Holocene warming periods^63^, following the opening of the Bering Strait. While this study suggests that climate warming may create favorable thermal conditions in the North Atlantic, where the gray whale was historically present, it remains uncertain if the eastern Pacific population will be able to establish a persistent population in the North Atlantic over a long time period. As suggested by^74^, the northern limit of the range of this species is only occupied during the summer months, and individuals following the traditional migratory routes may have limited movement through the Bering Strait. Then, the ongoing opening of the Northwest Passage, which is a consequence of climate warming, has created new opportunities for marine traffic and gas and oil activities, which may negatively impact gray whale migrations^73,74^. Overall, the gray whale and the Pacific right whale should be of particular concern for conservation prioritization given their high levels of vulnerability to climate change, their high functional originality and the current threats that they are facing.

For some species, such as the dugong or the walrus, the level of vulnerability differed between the RCP2.6 and the RCP8.5 scenarios. Both species were shown to be highly sensitive to climate change but not highly exposed, according to the RCP2.6 scenarios (see Appendices 6 and 7). In contrast, under the RCP8.5 high emission scenario, these two species would be intensively exposed to global warming and hence would be highly vulnerable (see Appendices 6 and 7). This result highlights that economic and political decisions towards the reduction of CO_2_ emissions and the mitigation of global warming can have serious consequences for the level of threat a species will face. This is particularly important because these species, while not classified in the top 20 most vulnerable species, were shown to be highly distinct in their evolutionary histories, in the case of the dugong (Fig. 2b), and in their traits, in the case of the walrus (Fig. 2c). Thus, the extinction of these species could provoke the loss of unique and important evolutionary lineages as well as a disruption in ecosystem functioning.

Among the top 20 most vulnerable species to climate change, some are still abundant within their geographical ranges and have shown no evidence of population declines, according to the IUCN. This is particularly the case for the spotted seal (*Phoca largha*) or the Pacific white-sided dolphin (*Lagenorhynchus obliquidens*), which are classified as least concern (Fig. 2a). Several species are also classified as data deficient, such as Baird’s beaked whale (*Berardius bairdii*). For these species, we recommend considering their potential vulnerabilities to climate change when setting their IUCN statuses. This is even more critical than the potential extinction of the most vulnerable species within the least concern IUCN category, which may lead to a disproportional loss of functional diversity (Fig. 4b) and could ultimately disrupt marine ecosystem functioning^75^. More generally, it would be particularly useful to consider the level of vulnerability to climate change when evaluating the IUCN statuses of marine mammals to implement mitigation actions on species that are not currently threatened (or have insufficient data) but that could be threatened in the near future^76^.

Scaling up our vulnerability analysis to the species assemblage level showed that, regardless of the RCP scenario, the North Pacific Ocean, Greenland Sea and Barents Sea (Fig. 3c,d) hosted the marine mammals that were most vulnerable to global warming. Indeed, these regions might face the strongest effects from global warming under both emissions scenarios (Fig. 3a,b) and have already undergone temperature increases 2–3 times higher than the changes to the global mean surface temperature over the past 150 years^1^. These regions should therefore be of particular concern for spatial monitoring for the conservation of marine mammals. Indeed, there are multiple threats to the marine mammals^77^ in the North Pacific Ocean, the Greenland Sea and the Barents Sea, such as marine traffic and offshore oil and gas exploitation that are known to impact cetaceans, such as through ocean noise pollution. These current threats could have additive or synergetic effects with climate change, which may therefore increase the overall vulnerability of marine mammals. In that context, future studies should focus on evaluating whether the combined effects of habitat loss, marine pollution and climate change are greater than the effects of each threat individually, which is challenging but crucial for providing effective conservation actions for marine mammals. In addition, these studies should consider the use of a finer spatial resolution to account for the species-habitat relationships (e.g.,^78^), which is required to evaluate the potential impacts of climate change on the regional spatial distributions of marine mammals.

Our results also suggested that the potential extinction of the marine mammals that are most vulnerable to global warming may lead to a disproportionate loss of functional diversity in the global marine mammal fauna (Fig. 4a); many of the most vulnerable species displaying a high level of functional originality (see Fig. S6). This projected loss of functional diversity may ultimately threaten marine ecosystem stability and service provisioning^33,75^. Similar findings have been reported for birds^77,79^ and marine fishes^80^ under climate change scenarios. These findings have a particular resonance in the context of the recent geological past. During the Plio-Pleistocene, large climatic oscillations and sea level changes caused numerous extinctions among the global marine megafauna, which directly induced an important loss of functional diversity that was not fully compensated by the evolution of new genera^81^. Marine megafauna have therefore been more sensitive to past environmental changes than previously assumed^82^, which implies that future climate change could pose a great challenge for large marine animals, especially mammals. However, our findings should be extended by downscaling our approach to a finer spatial resolution to assess the potential effects of climate change on the functional trait compositions of the local assemblages (for marine fishes^80^ and for birds^79^), which could have useful applications for prioritization in spatial conservation planning.

While the trait-based approach used in this study has the advantage of coupling the magnitude of climate change that a species would experience (exposure) with its intrinsic ability to tolerate changing climate (sensitivity), it nevertheless has some limitations. First, we did not consider the adaptive capacity of species to avoid the negative impacts of ocean warming through dispersal or microevolutionary changes^40,41^. However, the intrinsic and extrinsic abilities of marine mammals to colonize new favorable habitats and to avoid the impacts of ocean warming^11^ are poorly documented (but see^73^). For example, the population size that is often used as an indicator of adaptive capacity (i.e., species that are numerous should have more options for adapting or reestablishing themselves in local or new areas^25^) is not uniform among all marine mammals. We therefore decided to focus on the sensitivity and exposure dimensions to avoid biasing our assessment towards the most studied marine mammals. Nevertheless, we recognize that adaptive capacity is an important component of climate change vulnerability^40,41^, and we encourage future studies on marine mammals to focus on this issue. Second, when estimating exposure to global warming, we did not consider all components of the oceanic conditions under projected climate change scenarios, such as the loss of ice sea cover or changes in prey availability, which also influence the distributions of marine mammals^82^. We did not include the changes in Arctic sea ice cover because the CMIP5 models do not adequately represent the observed loss of Arctic sea ice cover over the last few decades, i.e., they largely underestimate this loss^83^. Our results should also be interpreted in the ligth of CMIP5 climate models that do not include the effects of meltwater from the ice sheets and ice shelves of Antarctica^84^. In addition, the projections of prey availability under climate change are currently not available at the global scale. We therefore encourage the development of climatic – biological coupled models, such as the “Model of Intermediate Complexity for Ecosystem Assessments” (MICE), which links krill and whale population dynamics with climate change drivers (i.e., changes in sea surface temperature, primary productivity and sea ice). For example, a MICE for the Southern Ocean was developed^82^ and showed that the projected declines in several whale species by 2100 could be the consequences of reduced prey from warming and increased interspecific competition between whale species. This modelling approach would complement to our trait-based approach by providing a mechanistic understanding of the vulnerabilities of various species to climate change.

## Supplementary Information

Supplementary Information.

## Data Availability

All traits data used to evaluate the species sensibilty to climate change along with presence absence matrix and the phylogenetical trees are available: https://figshare.com/articles/Input_data_for_Global_vulnerability_of_marine_mammals_to_global_warming_/11323304.

## References

[1] IPCC, Summary for Policymakers. In: IPCC Special Report on the Ocean and Cryosphere in a Changing Climate [H.-O. Pörtner, D. C. Roberts, V. Masson-Delmotte, P. Zhai, M. Tignor, E. Poloczanska, K. Mintenbeck, M. Nicolai, A. Okem, J. Petzold, B. Rama, N. Weyer (eds.)]. In press (2019).

[2] Cheng L, Abraham J, Hausfather Z, Trenberth KE (2019). How fast are the oceans warming?. Science..

[3] Lewandowska AM (2014). Effects of sea surface warming on marine plankton. Ecol. Lett..

[4] Faillettaz, R., Beaugrand, G., Goberville, E. & Kirby, R. R. Atlantic Multidecadal Oscillations drive the basin-scale distribution of Atlantic bluefin tuna. *Sci. Adv*. **5** (2019). 10.1126/sciadv.aar6993 PMC6314829 30613764

[5] Serpetti N (2017). Impact of ocean warming on sustainable fisheries management informs the Ecosystem Approach to Fisheries. Sci. Rep..

[6] Chambault P (2018). Sea surface temperature predicts the movements of an Arctic cetacean: the bowhead whale. Sci. Rep..

[7] Derville S (2019). Whales in warming water: Assessing breeding habitat diversity and adaptability in Oceania’s changing climate. Glob. Chang. Biol..

[8] Poff NL (2006). Functional trait niches of North American lotic insects: traits-based ecological applications in light of phylogenetic relationships. J. North Am. Benthol. Soc..

[9] Simmonds MP, Isaac SJ (2007). The impacts of climate change on marine mammals: early signs of significant problems. Oryx.

[10] Wild S (2019). Long-term decline in survival and reproduction of dolphins following a marine heatwave. Curr. Biol..

[11] Kaschner K, Tittensor DP, Ready J, Gerrodette T, Worm B (2011). Current and Future Patterns of Global Marine Mammal Biodiversity. PLoS One.

[12] Elliott, W. & Simmonds, M. Whales in Hot Water? The Impact of a Changing Climate on Whales, Dolphins and Porpoises: A call for action. *WWF-International, Gland Switz./WDCS, Chippenham, UK* (2007).

[13] Evans, P. G. H. & Bjørge, A. Impacts of climate change on marine biodiversity. *MCCIP Sci. Rev*. 134–148, 10.14465/2013.arc15.134-148 (2013).

[14] Kerosky SM (2012). Bryde’s whale seasonal range expansion and increasing presence in the Southern California Bight from 2000 to 2010. Deep Sea Res. Part I Oceanogr. Res. Pap..

[15] Schumann N, Gales NJ, Harcourt RG, Arnould JPY (2013). Impacts of climate change on Australian marine mammals. Aust. J. Zool..

[16] Harvell CD (2002). Climate warming and disease risks for terrestrial and marine biota. Science.

[17] Burek KA, Gulland FMD, O’Hara TM (2008). Effects of climate change on arctic marine mammal health. Ecol. Appl..

[18] Schipper J (2008). The Status of the World’s Land and Marine Mammals: Diversity, Threat, and Knowledge. Science..

[19] Davidson AD (2012). Drivers and hotspots of extinction risk in marine mammals. Proc. Natl. Acad. Sci. USA.

[20] Perrin, W. F., Würsig, B. G. & Thewissen, J. G. M. *Encyclopedia of marine mammals*. (Elsevier/Academic Press, 2009).

[21] Bowen WD (1997). Role of marine mammals in aquatic ecosystems. Mar. Ecol. Prog. Ser..

[22] Roman J, McCarthy JJ (2010). The Whale Pump: Marine Mammals Enhance Primary Productivity in a Coastal Basin. PLoS One.

[23] Berta, A. *Return to the Sea: The Life and Evolutionary Times of Marine Mammals*. (University of California Press, 2012).

[24] Estes JA (2011). Trophic downgrading of planet Earth. Science..

[25] Laidre KL (2008). Quantifying the sensitivity of Arctic marine mammals to climate-induced habitat change. Ecol. Appl..

[26] Moore SE, Huntington HP (2008). Arctic marine mammals and climate change: Impatcs and resiliance. Ecol. Appl..

[27] Pompa S, Ehrlich PR, Ceballos G (2011). Global distribution and conservation of marine mammals. Proc. Natl. Acad. Sci..

[28] Albouy C, Delattre VL, Mérigot B, Meynard CN, Leprieur F (2017). Multifaceted biodiversity hotspots of marine mammals for conservation priorities. Divers. Distrib..

[29] Dalongeville A, Andrello M, Mouillot D, Albouy C, Manel S (2015). Ecological traits shape genetic diversity patterns across the Mediterranean Sea: a quantitative review on fishes. J. Biogeogr..

[30] Mouillot D (2011). Protected and threatened components of fish biodiversity in the mediterranean sea. Curr. Biol..

[31] Devictor V (2010). Spatial mismatch and congruence between taxonomic, phylogenetic and functional diversity: the need for integrative conservation strategies in a changing world. Ecol. Lett..

[32] Guilhaumon François, Albouy Camille, Claudet Joachim, Velez Laure, Ben Rais Lasram Frida, Tomasini Jean-Antoine, Douzery Emmanuel J. P., Meynard Christine N., Mouquet Nicolas, Troussellier Marc, Araújo Miguel B., Mouillot David (2014). Representing taxonomic, phylogenetic and functional diversity: new challenges for Mediterranean marine-protected areas. Diversity and Distributions.

[33] Cadotte MW, Carscadden K, Mirotchnick N (2011). Beyond species: functional diversity and the maintenance of ecological processes and services. J. Appl. Ecol..

[34] Webb, C. O., Ackerly, D. D., McPeek, M. A. & Donoghue, M. J. Annual Review of Ecology and SystematicsPhylogenies and community ecology. **33**, 475–505 (2002).

[35] Diaz S, Cabido M (2001). Vive la difference: plant functional diversity matters to ecosystem processes. Trends Ecol. Evol..

[36] Mouillot D, Graham NAJ, Villéger S, Mason NWH, Bellwood DR (2013). A functional approach reveals community responses to disturbances. Trends Ecol. Evol..

[37] Webb CO, Ackerly DD, McPeek MA, Donoghue MJ (2002). Phylogenies and community ecology. Annu. Rev. Ecol. Syst..

[38] Maherali H, Klironomos JN (2007). Influence of Phylogeny on fungal community assembly and ecosystem functioning. Science..

[39] Mazel F (2018). Prioritizing phylogenetic diversity captures functional diversity unreliably. Nat. Commun..

[40] Foden WB (2013). Identifying the World’s Most Climate Change Vulnerable Species: A Systematic Trait-Based Assessment of all Birds, Amphibians and Corals. PLoS One.

[41] Foden WB (2019). Climate change vulnerability assessment of species. Wiley Interdiscip. Rev. Clim. Chang..

[42] Kaschner K, Watson R, Trites A, Pauly D (2006). Mapping world-wide distributions of marine mammal species using a relative environmental suitability (RES) model. Mar. Ecol. Prog. Ser..

[43] MacLeod C (2009). Global climate change, range changes and potential implications for the conservation of marine cetaceans: a review and synthesis. *Endanger*. Species Res..

[44] Fullard KJ (2000). Population structure of long-finned pilot whales in the North Atlantic: a correlation with sea surface temperature?. Mol. Ecol..

[45] Dickinson MG, Orme CDL, Suttle KB, Mace GM (2015). Separating sensitivity from exposure in assessing extinction risk from climate change. Sci. Rep..

[46] Böhm M (2016). Hot and bothered: Using trait-based approaches to assess climate change vulnerability in reptiles. Biol. Conserv..

[47] Conti L, Schmidt-Kloiber A, Grenouillet G, Graf W (2014). A trait-based approach to assess the vulnerability of European aquatic insects to climate change. Hydrobiologia.

[48] Hossain MA (2018). Assessing the vulnerability of freshwater crayfish to climate change. Divers. Distrib..

[49] H Rabinowitz, R. A. The Biological Aspects of Rare Plant Conservation Edited by Hugh Synge Seven forms of rarity. In *The Biological Aspects of* Rare Plant *Conservation* (ed. Hugh Syng) 205–217 (John Wiley & Sons, Ltd, 1981).

[50] Harnik PG, Simpson C, Payne JL (2012). Long-term differences in extinction risk among the seven forms of rarity. Proc. R. Soc. B Biol. Sci..

[51] Slatyer RA, Hirst M, Sexton JP (2013). Niche breadth predicts geographical range size: a general ecological pattern. Ecol. Lett..

[52] Thuiller W (2004). Patterns and uncertainties of species’ range shifts under climate change. Glob. Chang. Biol..

[53] Taylor KE, Stouffer RJ, Meehl GA (2012). An overview of CMIP5 and the experiment Design. Bull. Am. Meteorol. Soc..

[54] van Vuuren DP (2011). The representative concentration pathways: an overview. Clim. Change.

[55] Moss RH (2010). The next generation of scenarios for climate change research and assessment. Nature.

[56] Collins, M. *et al*. Long-term climate change: Projections, commitments and irreversibility, in Climate Change The Physical Science Basis. In *Contribution of Working Group I to the Fifth Assessment Report of the Intergovernmental Panel on* Climat (ed. Stocker *et al*. T. F.) (2013).

[57] Faith DPJ (1992). Conservation evaluation and phylogenetic diversity. Biol. Conserv..

[58] Fritz SA, Bininda-Emonds ORP, Purvis A (2009). Geographical variation in predictors of mammalian extinction risk: big is bad, but only in the tropics. Ecol. Lett..

[59] Kissling WD (2012). Towards novel approaches to modelling biotic interactions in multispecies assemblages at large spatial extents. J. Biogeogr..

[60] Villeger S, Mason NWH, Mouillot D (2008). New multidimensional functional diversity indices for a multifaceted framework in functional ecology. Ecology.

[61] Legendre, P. & Legendre, L. *Numerical Ecology* (1998).

[62] Maire E, Grenouillet G, Brosse S, Villéger S (2015). How many dimensions are needed to accurately assess functional diversity? A pragmatic approach for assessing the quality of functional spaces. Glob. Ecol. Biogeogr..

[63] Isaac NJB, Turvey ST, Collen B, Waterman C, Baillie JEM (2007). Mammals on the EDGE: Conservation Priorities Based on Threat and Phylogeny. PLoS One.

[64] Avila IC, Kaschner K, Dormann CF (2018). Current global risks to marine mammals: Taking stock of the threats. Biol. Conserv..

[65] IUCN. The IUCN Red List of Threatened Species. Version 2018-2 (2018).

[66] González-Suárez M, Gómez A, Revilla E (2013). Which intrinsic traits predict vulnerability to extinction depends on the actual threatening processes. Ecosphere.

[67] Tingley R, Hitchmough RA, Chapple DG (2013). Life-history traits and extrinsic threats determine extinction risk in New Zealand lizards. Biol. Conserv..

[68] Vasquez R, Simonetti JA (1999). Life history traits and sensitivity to landscape change: the case of birds and mammals of mediterranean Chile. Rev. Chil. Hist. Nat..

[69] Ivashchenko Y, Clapham P (2012). Soviet catches of right whales Eubalaena japonica and bowhead whales Balaena mysticetus in the North Pacific Ocean and the Okhotsk Sea. Endanger. Species Res..

[70] Josephson E, Smith TD, Reeves RR (2008). Historical distribution of right whales in the North Pacific. Fish Fish..

[71] Rugh DJ, Hobbs RC, Lerczak JA, Breiwick J (2005). Estimates of abundance of the eastern North Pacific stock of gray whales (Eschrichtius robustus) 1997–2002. J. Cetacean Res. Manag..

[72] Roman J (2014). Whales as marine ecosystem engineers. Front. Ecol. Environ..

[73] Alter SE (2015). Climate impacts on transocean dispersal and habitat in gray whales from the Pleistocene to 2100. Mol. Ecol..

[74] McLeod E, Salm R, Green A, Almany J (2009). Designing marine protected area networks to address the impacts of climate change. Front. Ecol. Environ..

[75] Frainer A (2017). Climate-driven changes in functional biogeography of Arctic marine fish communities. Proc. Natl. Acad. Sci. USA.

[76] Rosset V, Oertli B (2011). Freshwater biodiversity under climate warming pressure: Identifying the winners and losers in temperate standing waterbodies. Biol. Conserv..

[77] Thuiller W (2013). A road map for integrating eco-evolutionary processes into biodiversity models. Ecol. Lett..

[78] Gregr EJ (2011). Insights into North Pacific right whale Eubalaena Japonica habitat from historic whaling records. Endanger. Species Res..

[79] Barbet-Massin M, Jetz W (2015). The effect of range changes on the functional turnover, structure and diversity of bird assemblages under future climate scenarios. Glob. Chang. Biol..

[80] Albouy C (2015). Projected impacts of climate warming on the functional and phylogenetic components of coastal Mediterranean fish biodiversity. Ecography..

[81] Pimiento C (2017). The Pliocene marine megafauna extinction and its impact on functional diversity. Nat. Ecol. Evol..

[82] Tulloch VJD, Plagányi ÉE, Brown C, Richardson AJ, Matear R (2019). Future recovery of baleen whales is imperiled by climate change. Glob. Chang. Biol..

[83] Maslowski W, Clement Kinney J, Higgins M, Roberts A (2012). The Future of Arctic Sea Ice. Annu. Rev. Earth Planet. Sci..

[84] Bronselaer B (2018). Change in future climate due to Antarctic meltwater. Nature.

