# Author Correction: Global vulnerability of marine mammals to global warming

**DOI:** 10.1038/s41598-020-61227-4

**Published:** 2020-03-03

**Authors:** Camille Albouy, Valentine Delattre, Giulia Donati, Thomas L. Frölicher, Severine Albouy-Boyer, Marta Rufino, Loïc Pellissier, David Mouillot, Fabien Leprieur

**Affiliations:** 1 IFREMER, unité Ecologie et Modèles pour l’Halieutique, rue de l’Ile d’Yeu, BP21105, 44311 Nantes, cedex 3 France; 2 MARBEC, Univ Montpellier, CNRS, Ifremer, IRD, Montpellier, France; 3 Landscape Ecology, Institute of Terrestrial Ecosystems, ETH Zürich, 8092 Zürich, Switzerland; 4 Swiss Federal Research Institute WSL, 8903 Birmensdorf, Switzerland; 5 Climate and Environmental Physics, Physics Institute, University of Bern, Bern, Switzerland; 6 Oeschger Centre for Climate Change Research, University of Bern, Bern, Switzerland; 7 Cadres en Mission Nantes, Nantes, France; 8 MARE - Marine and Environmental Sciences Centre, Faculty of Sciences, University of Lisbon, Campo Grande, 1749-016 Lisboa Portugal; 9 CCMAR, The Centre of Marine Sciences, Universidade do Algarve, Campus de Gambelas, 8005-139 Faro, Portugal; 10 Institut Universitaire de France, Paris, France

Correction to: *Scientific Reports* 10.1038/s41598-019-57280-3, published online 17 January 2020

This Article contains errors in the legend of Figure 2.

In section (b):

“Each bubble is proportional to the level of functional originality (i.e., the measure of the extent to which a given species is functionally unique among the global marine mammal fauna).”

should read:

“Each bubble is proportional to the level of evolutionary distinctiveness (i.e., which represents the relative contribution of a species to the evolutionary history of the global marine mammal fauna).”

Additionally, in section (c):

“Each bubble is proportional to the level of evolutionary distinctiveness (i.e., which represents the relative contribution of a species to the evolutionary history of the global marine mammal fauna).”

should read:

“Each bubble is proportional to the level of functional originality (i.e., the measure of the extent to which a given species is functionally unique among the global marine mammal fauna).”

