# Supplementary material for: Global vulnerability of marine mammals to global warming
Source: Sci Rep. 2020 Jan 17;10:548. doi: 10.1038/s41598-019-57280-3 (PMC6969058; doi:10.1038/s41598-019-57280-3)
Supplement: Supplementary file 1 — Supplementary Information. [file 41598_2019_57280_MOESM1_ESM.pdf]

Supplementary Materials for

Global vulnerability of marine mammals to global warming

Camille Albouy, Valentine Delattre, Giulia Donati, Thomas Frölicher, Severine Albouy-Boyer, Marta Rufino, Loïc Pellissier, David Mouillot, Fabien Leprieur.

Correspondence to: albouycamille@gmail.com

This PDF file includes:

Supplementary method 1: Marine mammals traits description.

Supplementary method 2: Statistical sensitivity analysis for the sensitivity index.

Figs. S1 to S6

Tables S1 to S3

Supplementary method 1 | Marine mammals traits description.

Table S1 | Marine mammals species traits. Species traits considered to build the index of sensitivity to global warming. These fifteen traits cover five main categories and were selected to reflect the intrinsic sensitivity to global warming at the species-level. Each trait was divided into three ordered categories in order to quantify the degree to which a species may respond to global warming regarding its biological and ecological features. For each species, the sum of all trait values (between 0 and 2) resulted in an overall species-specific sensitivity ranking (see Methods).

Categories	**Trait**	***Modalities***		
		**0**	**1**	**2**
Feeding	Diet specialization	No prey cat. > 50%	At least one prey cat. ≥ 50%	One prey cat. ≥ 75%
	Habitat vertical specialization	all layers	benthic or mesopelagic	epipelagic
	Habitat horizontal specialization	all sections	shelf&slope or slope&offshore	shelf, slope or offshore
Habitat	Temperature range	[20;30]	[10;20[	[0;10[
	Temperature minimum	[16,7;25]	[8,3;16,7[	[0;8,3[
	Range area	[3,2;4,7]	[1,8;3,2[	[0,3;1,8[
	Range height	[99;160[	[25;99[	[0;25]
	Range fragmentation	1	[2;4]	]4;50[
	Ice concentration	[0;0.01]	0.5	[0.9;1]
Reproduction	Female sexual maturity	[2;6,3[	[6,3;10,7[	[10,7;15]
	Weaning	[40;60]	[20;40[	[0,1;20[
	Breeding site	offshore & coastal	land	ice or ice&land
	Inter-litter interval	[12;28[	[28;44[	[44;60]
Social behaviour	Social group size	[0,17;0,983[	[0,983;1,79[	[1,79;2,60]
Biology	Adult maximum body mass	[1,40;2,69[	[2,69;3,97[	[3,97;5,26]

1) **Diet specialization** is considered a fundamental variable because diet flexibility or ability to consume a variety of prey species should result in decreased sensitivity[1] and has been widely included in such analyses[1–4]. This trait was based on standardized diet composition among 9 prey categories (benthic invertebrates, large zooplankton, small squids, large squids, small pelagic fishes, mesopelagic fishes, miscellanous fishes, high vertebrates) available for 97 species of marine mammals[5]; missing values were filled in thereafter from scientific literature (e.g., seagrass eaters manatees *Trichechus spp.* and dugong *Dugong dugon* were categorized as specialist feeders). Ranking of diet specialization ranged between 0 (generalist

feeders, i.e. each prey category represents less than 50% of the diet) to 2 (highly specialized feeders, i.e. one prey category represents at least 75% of the diet).

2) **Habitat vertical specialization** is based on a four-modality nominal variable concerning habitat foraging preferences. This variable describes the capacity of the species to use different vertical layers as habitat. Indeed, habitat generalists are more buffered against global warming than habitat specialists because they can occupy a greater variety of habitats [1,6–8]. Species foraging in different habitats were considered as least sensitive (category 0), while those foraging in benthic or mesopelagic layers were noted as intermediate (category 1). Epipelagic feeders were considered the most sensitive ones (category 2).

3) **Habitat horizontal specialization** also concerns foraging preferences according to the distance from the coast. Reflecting habitat specialization, this variable has been widely used in studies upon vulnerability to global warming[1,6–9]. The geomorphological profile was divided into three sections, namely continental shelf, continental slope and offshore. Generalist feeders (i.e. foraging in every section) were noted as least sensitive, species foraging on two sections (both shelf and slope, or both slope and offshore) were intermediately sensitive and species foraging in only one section (either continental shelf, slope or offshore) were noted as most sensitive.

4) **Temperature range** is the difference between maximum and minimum Sea Surface Temperature (SST) encountered throughout the geographical range of a species. This variable is usually included in sensitivity indices regarding global warming amongst and across taxa[6–8,10]. Species encountering the most narrow temperature range were considered highly vulnerable while those encountering the widest range of SST were noted as least sensitive. Data were gathered from[11] for most species. Missing values were filled in *via* the Q-Gis 2.0.1 Dufour software by intersecting IUCN geographical range polygons (http://iucnredlist.org) and Bio-ORACLE raster environmental spatial data (http://www.oracle.ugent.be).

5) **Temperature minimum** describes the lowest value of SST met inside the geographical range of a species and is a classical variable in such analyses[6,12]. Lowest values reflect high sensitivity to global warming since their thermal requirements might not match environmental conditions anymore with the current and upcoming SST increase[13]. The source of the original data and the methodology to gather trait values were the same as the previous variable.

6) **Range area** is the total surface covered by the geographical range of a species at global scale and it is often used in this context[8,9,14,15]. Widely distributed species should be less vulnerable than narrowly distributed species given regional deviations in the direction and magnitude of climate warming[1,16]. According to the distribution of frequencies for this trait, this variable was log-transformed in order to attenuate the effect of highest values upon the lowest.

7) **Range height** is the difference between the highest latitude occupied by the geographical range of a species and the lowest one. This trait strongly influences the sensitivity of species to global warming[14,17]. In case of latitudinal discontinuous ranges (e.g. True’s beaked whale *Mesoplodon mirus*), the total range was decomposed into the sum of these separated areas. The range height was then approximated as the mean height of each of these areas.

8) **Range fragmentation** reflects the number of isolated areas occupied by a species within its global geographical range. Habitat fragmentation is thought to influence extinction and colonization of populations[18] and is known to have synergetic effects with global warming[19].

Species showing the highest range fragmentation were categorized as highly sensitive, while species with continuous range were considered as the least sensitive[14,17].

9) **Sea ice concentration** is a key environmental parameter that has been demonstrated to determine marine mammal species presence[20], since the edge of the pack ice represents an important feeding ground for many species[21]. The reduction or loss of seasonal ice cover associated with global warming has major implications for marine ecosystems. A progressive loss of ice cover would initially increase and eventually reduce or eliminate key habitats for marine mammals, e.g. polynya and ice edge habitats that are important areas for the exchange of energy between ecosystems. We compiled the data set from mean annual sea ice concentration data[22] (United States National Snow & Ice Data Center; NSIDC). Ice-dependent species were categorized as sensitive while ice-independent species (e.g. tropical species) were categorized as less sensitive.

10) **Female sexual maturity** is used as a proxy for speed of life history[23] and it is a reliable predictor of extinction risk across taxa as a proxy for Rmax[24]. This trait has been widely used in studies upon extinction risk or vulnerability to global warming, e.g. on mammals[17,24] and fish[3,14,24]. Sexual maturity is also a proxy for several key traits such as life expectancy, growth rate, predation exposure and ecosystem resilience[23]. The earliest mature species were categorized as the least sensitive to global warming. This trait has been considered on females rather than males because of the delay between sexual and social maturity in most Pinnipeds, rendering female sexual maturity more confident as a proxy for age at first reproduction.

11) **Weaning** is the mean duration of lactation. It is believed to reflect sensitivity to global warming as a predictor of growth rate[8,17]. Earliest weaning was linked to high sensitivity.

12) **Breeding site** describes the type of habitat used for mating. It reflects reliance on climate-sensitive habitats and has been used in similar studies[16]. Aquatic (coastal or offshore) mating species were considered as least sensitive. Land mating species were noted as intermediately sensitive. Highly sensitive species require ice habitat for breeding (either ice only or ice and land).

13) **Inter-litter interval** is the duration between two successive calving events. It is believed to be important in assessing the sensitivity to global warming as a proxy for both the reproductive effort[25] and the number of births per year[26]. The shorter is the inter-litter interval, the less sensitive is a species regarding to global warming since the resilience and resistance of the ecosystem is higher[23].

14) **Social group size** stands for either the harem size of Pinnipeds, the size of the migration group of Mysticetes, or the smallest stable group size of Odontocetes. This log-transformed variable is believed to play a role in assessing the extinction risk of marine mammals[26].

15) **Maximum body mass** was log-transformed prior to all analysis. This trait has been frequently used in extinction risk and vulnerability studies in a context of global warming[17,26] and can also be seen as a proxy for maximum body length that has also been widely used in this context[3,4,27]. The biggest species were considered as the most sensitive to global warming whereas the smallest ones were categorized as least sensitive. When not available, mean body mass was used instead and missing values were extrapolated from data on maximum body length through an allometric relationship.

Supplementary method 2 | Statistical sensitivity analysis for the sensitivity index.

For each quantitative trait, the initial categorization was calculated by using the tercile of the whole range of values (Fig. 1A). Four scenarios were performed by moving either the first break (a) or the second one (b) toward the minimum (scenarios a-d and b-d on Fig. 1B) or the maximum values (scenarios a+d and b+d; Fig. 1B). The amount (d) to which we moved the breaks varied between 1% and 33%. For each species, the sum of all trait values (between 0 and 2) resulted in an overall species-specific sensitivity ranking. Resulting values were then divided by the maximum sensitivity value in order to set the index between 0 (least sensitive species) and 1 (most sensitive species). Fig. 1B shows the correlation of Pearson between a given scenario and the initial categorization. Correlation ranging from 0.82 to 1 reflects that moving the breaks does not strongly impact the final results of sensitivity. These results show the robustness of our sensitivity index.

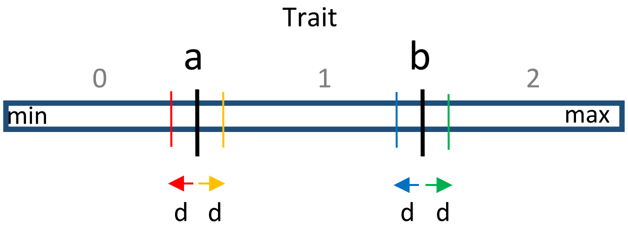

Fig. S1: methodology for the computing of the sensitivity analysis. For each trait, the initial categorization was calculated by cutting the whole range of values in three parts of equal range. The sensitivity analysis was performed by moving either the first break (a) or the second one (b) toward the minimum (-) or the maximum (+) values. The amount to which we moved the breaks (called d) varied between 1% and 33%.

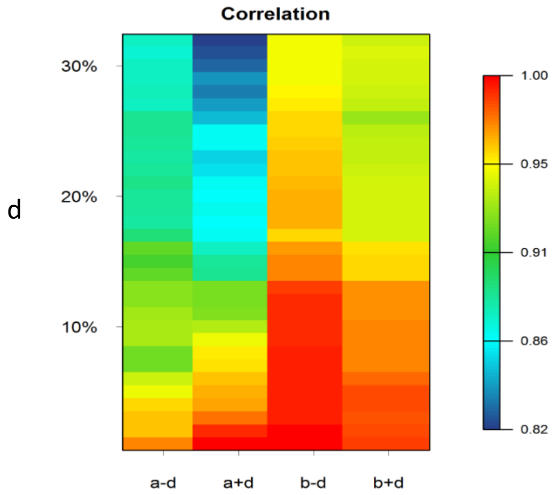

Fig. S2: correlation of Pearson between sensitivity resulting from each given scenario and the initial sensitivity results. X-axis shows the scenarios computed by moving either the lower break of categorization (a) or the upper one (b) towards the minimum (-d) or the maximum (+d) values. Y-axis shows the amount (d) to which we tested moving the breaks, from 1% to 33%.

Fig S3 | Projected mean sea surface temperature difference between the baseline period and the middle of the century and the associated marine mammals vulnerability.

Projected global warming was estimated using the mean sea surface temperature difference between the baseline period (1971-2000) and the middle of the century (2030-2059) according to the RCP2.6 (a) and RCP8.5 (b) scenarios for 11 different CMIP5 Earth system models (MRI-CGCM3, IPSL-CM5A-LR, GFDL-ESM2G, GFDL-ESM2M, IPSL-CM5A-MR, MIROC-ESM, MPI-ESM-LR, GFDL-CM3, CSIRO, CanESM2). Assemblage-level vulnerability of marine mammals to global warming for the period (2070-2099) based on (c) the emission mitigation scenario with a net radiative forcing by year 2100 of 2.6 W/m² (RCP2.6) and (d) the high-carbon emission, business as usual scenario with a net radiative forcing by year 2100 of 8.5 W/m² (RCP8.5). To evaluate the vulnerability of marine mammals to global warming at the assemblage level, we averaged the vulnerability of each species occurring in a grid cell (1° x 1° grid-cells, ~10,000-km²). Maps were done using R 3.6.0 software (https://www.r-project.org/).

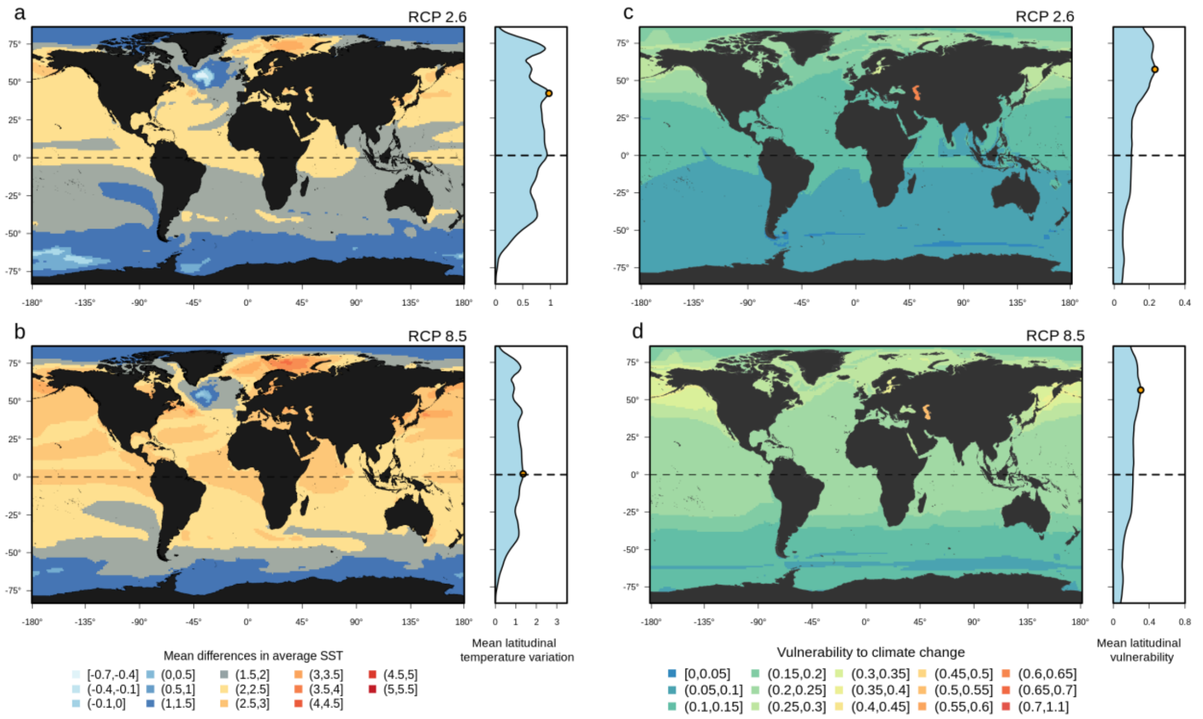

Fig. S4 | Sensitivity exposure and vulnerability to global warming for marine mammals: Values distribution of (a) the marine mammals' sensitivity to global warming, (b) the exposure to global warming according to the RCP2.6 and RCP8.5 scenarios and time periods (2030-2059, 2070-2099), (c) the vulnerability exposure to global warming according to both RCPs scenarios and time periods (2030-2059, 2070-2099).

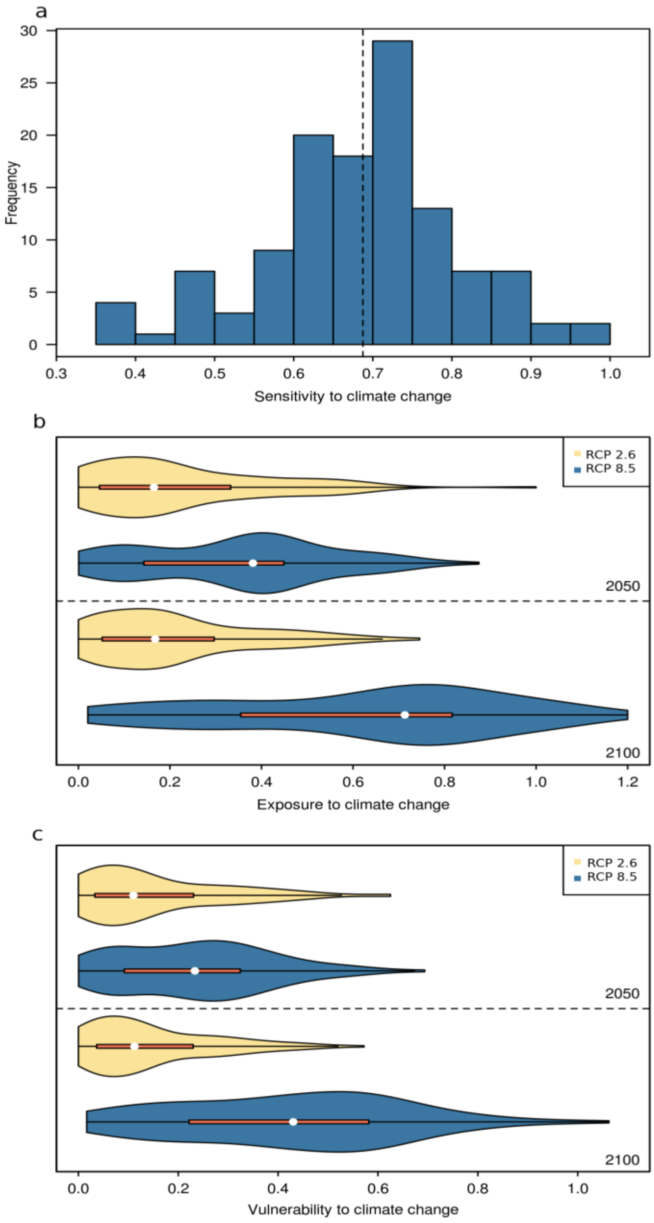

Table S2 | Ranking of marine mammals according to their vulnerability to global warming. Here, we present the results for the period (2030-2059) and for the emissions scenarios RCP2.6 and RCP8.5. The IUCN extinction risk categories CR, EN, VU, NT, LC and DD respectively refer to Critically Endangered, Endangered, Vulnerable, Near Threatened, Least Concern and Data Deficient. Vulnerability index is the product of intrinsic sensitivity to global warming by exposure. Sensitivity was based on traits reflecting the degree to which a species may respond to global warming regarding its bio-ecological features. Exposure quantifies the actual change in temperature that a species is likely to face within its geographical range. We evaluated these changes using 11 different CMIP5 Earth system models (MRI-CGCM3, IPSL-CM5A-LR, GFDL-ESM2G, GFDL-ESM2M, IPSL-CM5A-MR, MIROC-ESM, MPI-ESM-LR, GFDL-CM3, CSIRO, CanESM2).

RCP26						RCP85				
Species	Abreviation	IUCN status	Sensibility	Exposure	Vulnerability	Species	IUCN status	Sensibility	Exposure	Vulnerability
Pusa caspica	P.casp	EN	0.63	1.00	0.63	Eubalaena japonica	EN	1.00	0.69	0.69
Eubalaena japonica	E.japo	EN	1.00	0.62	0.62	Eschrichtius robustus	LC	0.92	0.63	0.58
Berardius bairdii	B.bair	DD	0.88	0.55	0.48	Berardius bairdii	DD	0.88	0.63	0.56
Eschrichtius robustus	E.robu	LC	0.92	0.51	0.47	Pusa caspica	EN	0.63	0.88	0.55
Mesoplodon stejnegeri	M.stej	DD	0.73	0.62	0.45	Lagenorhynchus obliquidens	LC	0.79	0.66	0.53
Phoca largha	P.larg	LC	0.75	0.59	0.44	Mesoplodon stejnegeri	DD	0.73	0.72	0.53
Lagenorhynchus obliquidens	L.obli	LC	0.79	0.55	0.43	Phoca largha	LC	0.75	0.68	0.51
Enhydra lutris	E.lutr	EN	0.63	0.64	0.40	Lissodelphis borealis	LC	0.75	0.66	0.49
Lissodelphis borealis	L.bore	LC	0.75	0.53	0.40	Phocoenoides dalli	LC	0.71	0.64	0.45
Phocoenoides dalli	P.dall	LC	0.71	0.56	0.40	Enhydra lutris	EN	0.63	0.72	0.45
Mirounga angustirostris	M.angu	LC	0.75	0.50	0.38	Neophocaena asiaeorientalis	EN	0.73	0.60	0.44
Neophocaena asiaeorientalis	N.asia	EN	0.73	0.51	0.37	Halichoerus grypus	LC	0.71	0.61	0.43
Balaena mysticetus	B.myst	LC	0.88	0.41	0.36	Mirounga angustirostris	LC	0.75	0.56	0.42
Halichoerus grypus	H.gryp	LC	0.71	0.50	0.36	Eumetopias jubatus	NT	0.58	0.70	0.41
Eumetopias jubatus	E.juba	NT	0.58	0.61	0.35	Balaena mysticetus	LC	0.88	0.47	0.41
Callorhinus ursinus	C.ursi	VU	0.58	0.58	0.34	Callorhinus ursinus	VU	0.58	0.69	0.40
Lagenorhynchus albirostris	L.albi	LC	0.77	0.41	0.32	Arctocephalus galapagoensis	EN	0.67	0.60	0.40
Odobenus rosmarus	O.rosm	VU	1.00	0.31	0.31	Zalophus wollebaeki	EN	0.67	0.60	0.40
Phocoena phocoena	P.phoc	LC	0.63	0.49	0.31	Phoca vitulina	LC	0.79	0.49	0.38
Histriophoca fasciata	H.fasc	LC	0.58	0.52	0.31	Lagenorhynchus albirostris	LC	0.77	0.50	0.38
Phoca vitulina	P.vitu	LC	0.79	0.38	0.30	Phocoena phocoena	LC	0.63	0.61	0.38
Eubalaena glacialis	E.glac	EN	0.88	0.33	0.29	Monachus monachus	EN	0.63	0.58	0.37
Cystophora cristata	C.cris	VU	0.75	0.38	0.28	Eubalaena glacialis	EN	0.88	0.42	0.36
Monachus monachus	M.mona	EN	0.63	0.45	0.28	Trichechus manatus	VU	0.75	0.48	0.36
Erignathus barbatus	E.barb	LC	0.85	0.32	0.28	Dugong dugon	VU	0.88	0.40	0.35
Mesoplodon carlhubbsi	M.carl	DD	0.63	0.43	0.27	Histriophoca fasciata	LC	0.58	0.59	0.34
Delphinapterus leucas	D.leuc	LC	0.79	0.34	0.27	Hyperoodon ampullatus	DD	0.79	0.43	0.34
Hyperoodon ampullatus	H.ampu	DD	0.79	0.33	0.26	Cystophora cristata	VU	0.75	0.45	0.34
Lagenorhynchus acutus	L.acut	LC	0.63	0.40	0.25	Odobenus rosmarus	VU	1.00	0.34	0.34
Mesoplodon bidens	M.bide	DD	0.71	0.34	0.24	Mesoplodon perrini	DD	0.63	0.54	0.34
Arctocephalus galapagoensis	A.gala	EN	0.67	0.35	0.23	Zalophus californianus	LC	0.67	0.49	0.32
Phocoena sinus	P.sinu	CR	0.63	0.36	0.23	Erignathus barbatus	LC	0.85	0.38	0.32
Pagophilus groenlandicus	P.groe	LC	0.63	0.36	0.22	Mesoplodon carlhubbsi	DD	0.63	0.51	0.32
Zalophus californianus	Z.cali	LC	0.67	0.33	0.22	Trichechus senegalensis	VU	0.79	0.41	0.32
Mesoplodon perrini	M.perr	DD	0.63	0.34	0.21	Stenella clymene	DD	0.71	0.45	0.32
Zalophus wollebaeki	Z.woll	EN	0.67	0.30	0.20	Grampus griseus	LC	0.77	0.41	0.31
Trichechus manatus	T.mana	VU	0.75	0.26	0.19	Sousa teuszii	CR	0.69	0.45	0.31
Delphinus delphis	D.delp	LC	0.63	0.29	0.18	Lagenodelphis hosei	LC	0.75	0.41	0.31
Pusa hispida	P.hisp	LC	0.83	0.21	0.18	Delphinapterus leucas	LC	0.79	0.38	0.30
Arctocephalus townsendi	A.town	LC	0.67	0.25	0.17	Monachus schauinslandi	EN	0.73	0.41	0.30
Grampus griseus	G.gris	LC	0.77	0.21	0.16	Peponocephala electra	LC	0.75	0.40	0.30
Stenella clymene	S.clym	DD	0.71	0.22	0.16	Indopacetus pacificus	DD	0.67	0.45	0.30
Monachus schauinslandi	M.scha	EN	0.73	0.20	0.15	Tursiops aduncus	DD	0.73	0.41	0.30
Ursus maritimus	U.mari	VU	0.83	0.18	0.15	Mesoplodon bidens	DD	0.71	0.42	0.30
Delphinus capensis	D.cape	DD	0.67	0.20	0.13	Lagenorhynchus acutus	LC	0.63	0.47	0.29
Stenella frontalis	S.fron	DD	0.54	0.25	0.13	Balaenoptera omurai	DD	0.71	0.41	0.29
Sotalia guianensis	S.guia	DD	0.63	0.20	0.13	Delphinus capensis	DD	0.67	0.43	0.28
Balaenoptera physalus	B.phys	EN	0.75	0.16	0.12	Delphinus delphis	LC	0.63	0.45	0.28
Lagenodelphis hosei	L.hose	LC	0.75	0.16	0.12	Pagophilus groenlandicus	LC	0.63	0.45	0.28
Balaenoptera musculus	B.musc	EN	0.79	0.15	0.12	Sotalia guianensis	DD	0.63	0.43	0.27
Indopacetus pacificus	I.paci	DD	0.67	0.18	0.12	Stenella attenuata	LC	0.71	0.38	0.27
Sousa teuszii	S.teus	CR	0.69	0.18	0.12	Steno bredanensis	LC	0.67	0.39	0.26
Physeter macrocephalus	P.macr	VU	0.73	0.16	0.12	Arctocephalus townsendi	LC	0.67	0.39	0.26
Trichechus senegalensis	T.sene	VU	0.79	0.15	0.12	Stenella longirostris	DD	0.67	0.39	0.26
Mesoplodon mirus	M.miru	DD	0.77	0.15	0.12	Phocoena sinus	CR	0.63	0.42	0.26
Peponocephala electra	P.elec	LC	0.75	0.16	0.12	Globicephala macrorhynchus	DD	0.67	0.38	0.25
Mesoplodon europaeus	M.euro	DD	0.50	0.24	0.12	Sousa chinensis	VU	0.60	0.41	0.25
Steno bredanensis	S.bred	LC	0.67	0.17	0.11	Orcaella brevirostris	EN	0.65	0.38	0.24

Table S2

RCP26						RCP85				
Balaenoptera borealis	B.bore	EN	0.71	0.16	0.11	Stenella frontalis	DD	0.54	0.43	0.24
Balaenoptera omurai	B.omur	DD	0.71	0.16	0.11	Orcaella heinsohni	VU	0.58	0.40	0.23
Orcinus orca	O.orca	DD	0.67	0.17	0.11	Pseudorca crassidens	DD	0.58	0.40	0.23
Dugong dugon	D.dugo	VU	0.88	0.13	0.11	Neophocaena phocaenoides	VU	0.58	0.40	0.23
Stenella longirostris	S.long	DD	0.67	0.16	0.11	Balaenoptera musculus	EN	0.79	0.29	0.23
Stenella attenuata	S.atte	LC	0.71	0.15	0.11	Mesoplodon peruvianus	DD	0.54	0.42	0.23
Globicephala macrorhynchus	G.macr	DD	0.67	0.16	0.11	Cephalorhynchus heavisidii	DD	0.75	0.30	0.23
Tursiops aduncus	T.adun	DD	0.73	0.14	0.11	Balaenoptera physalus	EN	0.75	0.30	0.23
Pseudorca crassidens	P.cras	DD	0.58	0.18	0.10	Physeter macrocephalus	VU	0.73	0.30	0.22
Megaptera novaeangliae	M.nova	LC	0.58	0.17	0.10	Mesoplodon mirus	DD	0.77	0.29	0.22
Cephalorhynchus heavisidii	C.heav	DD	0.75	0.12	0.09	Pusa hispida	LC	0.83	0.26	0.22
Stenella coeruleoalba	S.coer	LC	0.50	0.17	0.09	Balaenoptera borealis	EN	0.71	0.30	0.21
Mesoplodon peruvianus	M.peru	DD	0.54	0.16	0.08	Mesoplodon europaeus	DD	0.50	0.43	0.21
Neophocaena phocaenoides	N.phoc	VU	0.58	0.14	0.08	Mesoplodon ginkgodens	DD	0.54	0.39	0.21
Balaenoptera edeni	B.eden	DD	0.50	0.17	0.08	Orcinus orca	DD	0.67	0.30	0.20
Arctocephalus pusillus	A.pusi	LC	0.67	0.12	0.08	Feresa attenuata	DD	0.50	0.38	0.19
Mesoplodon ginkgodens	M.gink	DD	0.54	0.15	0.08	Balaenoptera edeni	DD	0.50	0.38	0.19
Tursiops truncatus	T.trun	LC	0.48	0.17	0.08	Stenella coeruleoalba	LC	0.50	0.37	0.19
Feresa attenuata	F.atte	DD	0.50	0.16	0.08	Megaptera novaeangliae	LC	0.58	0.30	0.18
Sousa chinensis	S.chin	VU	0.60	0.13	0.08	Arctocephalus pusillus	LC	0.67	0.26	0.18
Ziphius cavirostris	Z.cavi	LC	0.42	0.19	0.08	Tursiops truncatus	LC	0.48	0.37	0.18
Mesoplodon densirostris	M.dens	DD	0.46	0.17	0.08	Mesoplodon densirostris	DD	0.46	0.37	0.17
Monodon monoceros	M.mono	LC	0.88	0.08	0.07	Ursus maritimus	VU	0.83	0.20	0.16
Globicephala melas	G.mela	DD	0.75	0.10	0.07	Ziphius cavirostris	LC	0.42	0.37	0.15
Kogia breviceps	K.brev	DD	0.38	0.17	0.07	Kogia breviceps	DD	0.38	0.37	0.14
Orcaella brevirostris	O.brev	EN	0.65	0.10	0.06	Kogia sima	DD	0.38	0.37	0.14
Kogia sima	K.sima	DD	0.38	0.16	0.06	Cephalorhynchus hectori	EN	0.75	0.18	0.13
Balaenoptera acutorostrata	B.acut	LC	0.35	0.17	0.06	Pontoporia blainvillei	VU	0.67	0.19	0.13
Orcaella heinsohni	O.hein	VU	0.58	0.10	0.06	Globicephala melas	DD	0.75	0.17	0.13
Pontoporia blainvillei	P.blai	VU	0.67	0.07	0.05	Monodon monoceros	LC	0.88	0.14	0.13
Arctocephalus tropicalis	A.trop	LC	0.63	0.06	0.04	Balaenoptera acutorostrata	LC	0.35	0.30	0.11
Tasmacetus shepherdi	T.shep	DD	0.81	0.04	0.03	Lagenorhynchus obscurus	DD	0.67	0.15	0.10
Balaenoptera bonaerensis	B.bona	DD	0.69	0.05	0.03	Balaenoptera bonaerensis	DD	0.69	0.14	0.09
Eubalaena australis	E.aust	LC	0.79	0.04	0.03	Arctocephalus tropicalis	LC	0.63	0.15	0.09
Lagenorhynchus obscurus	L.obsc	DD	0.67	0.05	0.03	Tasmacetus shepherdi	DD	0.81	0.11	0.09
Lobodon carcinophaga	L.carc	LC	0.92	0.03	0.03	Caperea marginata	DD	0.75	0.11	0.08
Berardius arnuxii	B.arnu	DD	0.83	0.04	0.03	Mesoplodon hectori	DD	0.63	0.13	0.08
Hyperoodon planifrons	H.plan	LC	0.83	0.04	0.03	Arctocephalus forsteri	LC	0.63	0.12	0.08
Caperea marginata	C.marg	DD	0.75	0.04	0.03	Eubalaena australis	LC	0.79	0.09	0.07
Mesoplodon bowdoini	M.bowd	DD	0.63	0.05	0.03	Otaria flavescens	LC	0.67	0.11	0.07
Mesoplodon hectori	M.hect	DD	0.63	0.04	0.03	Lontra felina	EN	0.56	0.12	0.07
Ommatophoca rossii	O.ross	LC	0.81	0.03	0.03	Mesoplodon bowdoini	DD	0.63	0.11	0.07
Lissodelphis peronii	L.pero	DD	0.71	0.04	0.03	Lissodelphis peronii	DD	0.71	0.10	0.07
Mesoplodon layardii	M.laya	DD	0.63	0.04	0.03	Phocoena spinipinnis	DD	0.50	0.13	0.07
Lagenorhynchus cruciger	L.cruc	LC	0.71	0.03	0.02	Berardius arnuxii	DD	0.83	0.08	0.07
Mirounga leonina	M.leon	LC	0.71	0.03	0.02	Mesoplodon traversii	DD	0.63	0.10	0.06
Leptonychotes weddellii	L.wedd	LC	0.85	0.03	0.02	Mesoplodon layardii	DD	0.63	0.10	0.06
Arctocephalus forsteri	A.fors	LC	0.63	0.03	0.02	Hyperoodon planifrons	LC	0.83	0.07	0.06
Cephalorhynchus hectori	C.hect	EN	0.75	0.03	0.02	Arctocephalus australis	LC	0.67	0.09	0.06
Mesoplodon grayi	M.gray	DD	0.58	0.03	0.02	Neophoca cinerea	EN	0.38	0.14	0.05
Phocoena dioptrica	P.diop	DD	0.63	0.03	0.02	Mesoplodon grayi	DD	0.58	0.08	0.05
Otaria flavescens	O.flav	LC	0.67	0.03	0.02	Lagenorhynchus cruciger	LC	0.71	0.06	0.04
Hydrurga leptonyx	H.lept	LC	0.83	0.02	0.02	Phocoena dioptrica	DD	0.63	0.06	0.04
Phocoena spinipinnis	P.spin	DD	0.50	0.03	0.02	Mirounga leonina	LC	0.71	0.05	0.04
Arctocephalus australis	A.aust	LC	0.67	0.02	0.01	Lobodon carcinophaga	LC	0.92	0.03	0.03
Mesoplodon traversii	M.trav	DD	0.63	0.02	0.01	Ommatophoca rossii	LC	0.81	0.03	0.02
Lontra felina	L.feli	EN	0.56	0.02	0.01	Leptonychotes weddellii	LC	0.85	0.03	0.02
Arctocephalus gazella	A.gaze	LC	0.69	0.01	0.01	Hydrurga leptonyx	LC	0.83	0.02	0.02
Arctocephalus philippii	A.phil	LC	0.79	0.00	0.00	Arctocephalus gazella	LC	0.69	0.02	0.01
Cephalorhynchus commersonii	C.comm	LC	0.79	0.00	0.00	Arctocephalus philippii	LC	0.79	0.01	0.01
Cephalorhynchus eutropia	C.eutr	NT	0.79	0.00	0.00	Cephalorhynchus eutropia	NT	0.79	0.00	0.00
Lagenorhynchus australis	L.aust	DD	0.75	0.00	0.00	Lagenorhynchus australis	DD	0.75	0.00	0.00
Neophoca cinerea	N.cine	EN	0.38	0.00	0.00	Cephalorhynchus commersonii	LC	0.79	0.00	0.00
Phocarctos hookeri	P.hook	EN	0.71	0.00	0.00	Phocarctos hookeri	EN	0.71	0.00	0.00

Table S3 | Ranking of marine mammals according to their vulnerability to global warming. Here, we present the results for the period (2070-2099) and for the emissions scenarios RCP2.6 and RCP8.5. The IUCN extinction risk categories CR, EN, VU, NT, LC and DD respectively refer to Critically Endangered, Endangered, Vulnerable, Near Threatened, Least Concern and Data Deficient. Vulnerability index is the product of intrinsic sensitivity to global warming by exposure. Sensitivity was based on traits reflecting the degree to which a species may respond to global warming regarding its bio-ecological features. Exposure quantifies the actual change in temperature that a species is likely to face within its geographical range. We evaluated these changes using 11 different CMIP5 Earth system models (MRI-CGCM3, IPSL-CM5A-LR, GFDL-ESM2G, GFDL-ESM2M, IPSL-CM5A-MR, MIROC-ESM, MPI-ESM-LR, GFDL-CM3, CSIRO, CanESM2).

RCP26						RCP85				
Species	Abbreviation	IUCN status	Sensibility	Exposure	Vulnerability	Species	IUCN status	Sensibility	Exposure	Vulnerability
Eubalaena japonica	E.japo	EN	1.00	0.57	0.57	Eubalaena japonica	EN	1.00	1.06	1.06
Eschrichtius robustus	E.robu	LC	0.92	0.52	0.47	Eschrichtius robustus	LC	0.92	1.03	0.94
Pusa caspica	P.casp	EN	0.63	0.75	0.47	Berardius bairdii	DD	0.88	1.03	0.90
Mesoplodon stejnegeri	M.stej	DD	0.73	0.61	0.45	Lagenorhynchus obliquidens	LC	0.79	1.03	0.81
Berardius bairdii	B.bair	DD	0.88	0.49	0.43	Phoca largha	LC	0.75	1.07	0.80
Lagenorhynchus obliquidens	L.obli	LC	0.79	0.51	0.40	Mesoplodon stejnegeri	DD	0.73	1.06	0.77
Phoca largha	P.larg	LC	0.75	0.53	0.40	Lissodelphis borealis	LC	0.75	1.02	0.77
Enhydra lutris	E.lutr	EN	0.63	0.64	0.40	Neophocaena asiaeorientalis	EN	0.73	1.05	0.76
Eumetopias jubatus	E.juba	NT	0.58	0.61	0.35	Pusa caspica	EN	0.63	1.20	0.75
Phocoenoides dalli	P.dall	LC	0.71	0.50	0.35	Phocoenoides dalli	LC	0.71	1.03	0.73
Lissodelphis borealis	L.bore	LC	0.75	0.46	0.34	Mirounga angustirostris	LC	0.75	0.96	0.72
Mirounga angustirostris	M.angu	LC	0.75	0.45	0.34	Odobenus rosmarus	VU	1.00	0.70	0.70
Neophocaena asiaeorientalis	N.asia	EN	0.73	0.44	0.32	Dugong dugon	VU	0.88	0.77	0.67
Lagenorhynchus albirostris	L.albi	LC	0.77	0.41	0.32	Balaena mysticetus	LC	0.88	0.75	0.66
Callorhinus ursinus	C.ursi	VU	0.58	0.55	0.32	Enhydra lutris	EN	0.63	1.04	0.65
Halichoerus grypus	H.gryp	LC	0.71	0.45	0.32	Monachus monachus	EN	0.63	1.03	0.64
Balaena mysticetus	B.myst	LC	0.88	0.34	0.30	Arctocephalus galapagoensis	EN	0.67	0.96	0.64
Phocoena phocoena	P.phoc	LC	0.63	0.47	0.29	Trichechus senegalensis	VU	0.79	0.81	0.64
Zalophus californianus	Z.cali	LC	0.67	0.43	0.29	Monachus schauinslandi	EN	0.73	0.88	0.64
Phoca vitulina	P.vitu	LC	0.79	0.36	0.28	Zalophus wollebaeki	EN	0.67	0.95	0.64
Cystophora cristata	C.cris	VU	0.75	0.36	0.27	Trichechus manatus	VU	0.75	0.85	0.63
Histriophoca fasciata	H.fasc	LC	0.58	0.45	0.26	Eumetopias jubatus	NT	0.58	1.06	0.62
Eubalaena glacialis	E.glac	EN	0.88	0.30	0.26	Callorhinus ursinus	VU	0.58	1.05	0.61
Monachus monachus	M.mona	EN	0.63	0.41	0.26	Zalophus californianus	LC	0.67	0.91	0.61
Odobenus rosmarus	O.rosm	VU	1.00	0.25	0.25	Mesoplodon carlhubbsi	DD	0.63	0.97	0.60
Lagenorhynchus acutus	L.acut	LC	0.63	0.39	0.25	Stenella clymene	DD	0.71	0.84	0.59
Hyperoodon ampullatus	H.ampu	DD	0.79	0.31	0.24	Phoca vitulina	LC	0.79	0.75	0.59
Erignathus barbatus	E.barb	LC	0.85	0.29	0.24	Grampus griseus	LC	0.77	0.76	0.59
Arctocephalus galapagoensis	A.gala	EN	0.67	0.36	0.24	Sousa teuszii	CR	0.69	0.85	0.58
Zalophus wollebaeki	Z.woll	EN	0.67	0.35	0.23	Lagenodelphis hosei	LC	0.75	0.78	0.58
Delphinapterus leucas	D.leuc	LC	0.79	0.29	0.23	Arctocephalus townsendi	LC	0.67	0.87	0.58
Mesoplodon carlhubbsi	M.carl	DD	0.63	0.36	0.22	Mesoplodon perrini	DD	0.63	0.92	0.58
Mesoplodon bidens	M.bide	DD	0.71	0.31	0.22	Tursiops aduncus	DD	0.73	0.79	0.58
Pagophilus groenlandicus	P.groe	LC	0.63	0.35	0.22	Peponocephala electra	LC	0.75	0.77	0.58
Monachus schauinslandi	M.scha	EN	0.73	0.26	0.19	Halichoerus grypus	LC	0.71	0.81	0.57
Delphinus delphis	D.delp	LC	0.63	0.27	0.17	Indopacetus pacificus	DD	0.67	0.85	0.56
Trichechus senegalensis	T.sene	VU	0.79	0.21	0.17	Delphinapterus leucas	LC	0.79	0.71	0.56
Stenella clymene	S.clym	DD	0.71	0.23	0.16	Phocoena sinus	CR	0.63	0.89	0.56
Trichechus manatus	T.mana	VU	0.75	0.21	0.16	Delphinus capensis	DD	0.67	0.83	0.56
Grampus griseus	G.gris	LC	0.77	0.20	0.16	Balaenoptera omurai	DD	0.71	0.77	0.55
Sousa teuszii	S.teus	CR	0.69	0.22	0.15	Histriophoca fasciata	LC	0.58	0.94	0.55
Pusa hispida	P.hisp	LC	0.83	0.18	0.15	Erignathus barbatus	LC	0.85	0.64	0.54
Mesoplodon perrini	M.perr	DD	0.63	0.24	0.15	Phocoena phocoena	LC	0.63	0.86	0.54
Delphinus capensis	D.cape	DD	0.67	0.21	0.14	Eubalaena glacialis	EN	0.88	0.60	0.53
Sotalia guianensis	S.guia	DD	0.63	0.22	0.14	Cephalorhynchus heavisidii	DD	0.75	0.70	0.52
Lagenodelphis hosei	L.hose	LC	0.75	0.18	0.13	Lagenorhynchus albirostris	LC	0.77	0.68	0.52
Arctocephalus townsendi	A.town	LC	0.67	0.20	0.13	Stenella attenuata	LC	0.71	0.74	0.52
Stenella frontalis	S.fron	DD	0.54	0.24	0.13	Hyperoodon ampullatus	DD	0.79	0.65	0.51
Indopacetus pacificus	I.paci	DD	0.67	0.19	0.13	Delphinus delphis	LC	0.63	0.82	0.51
Peponocephala electra	P.elec	LC	0.75	0.17	0.13	Stenella longirostris	DD	0.67	0.77	0.51
Phocoena sinus	P.sinu	CR	0.63	0.20	0.13	Steno bredanensis	LC	0.67	0.76	0.51
Balaenoptera physalus	B.phys	EN	0.75	0.17	0.12	Globicephala macrorhynchus	DD	0.67	0.74	0.49
Balaenoptera musculus	B.musc	EN	0.79	0.16	0.12	Sousa chinensis	VU	0.60	0.79	0.48
Physeter macrocephalus	P.macr	VU	0.73	0.17	0.12	Cystophora cristata	VU	0.75	0.63	0.47
Steno bredanensis	S.bred	LC	0.67	0.18	0.12	Sotalia guianensis	DD	0.63	0.75	0.47
Stenella attenuata	S.atte	LC	0.71	0.16	0.11	Neophocaena phocaenoides	VU	0.58	0.80	0.47
Mesoplodon peruvianus	M.peru	DD	0.54	0.21	0.11	Orcaella brevirostris	EN	0.65	0.72	0.46
Stenella longirostris	S.long	DD	0.67	0.17	0.11	Orcaella heinsohni	VU	0.58	0.79	0.46

Table S3

RCP26						RCP85				
Mesoplodon europaeus	M.euro	DD	0.50	0.23	0.11	Pseudorca crassidens	DD	0.58	0.77	0.45
Balaenoptera borealis	B.bore	EN	0.71	0.16	0.11	Mesoplodon peruvianus	DD	0.54	0.81	0.44
Ursus maritimus	U.mari	VU	0.83	0.14	0.11	Mesoplodon bidens	DD	0.71	0.61	0.43
Globicephala macrorhynchus	G.macr	DD	0.67	0.17	0.11	Balaenoptera musculus	EN	0.79	0.54	0.43
Orcinus orca	O.orca	DD	0.67	0.17	0.11	Cephalorhynchus hectori	EN	0.75	0.57	0.43
Mesoplodon mirus	M.miru	DD	0.77	0.14	0.11	Stenella frontalis	DD	0.54	0.79	0.43
Pseudorca crassidens	P.cras	DD	0.58	0.19	0.11	Pagophilus groenlandicus	LC	0.63	0.68	0.42
Cephalorhynchus heavisidii	C.heav	DD	0.75	0.14	0.10	Balaenoptera physalus	EN	0.75	0.56	0.42
Balaenoptera omurai	B.omur	DD	0.71	0.14	0.10	Mesoplodon ginkgodens	DD	0.54	0.77	0.42
Megaptera novaeangliae	M.nova	LC	0.58	0.17	0.10	Mesoplodon mirus	DD	0.77	0.53	0.41
Neophocaena phocaenoides	N.phoc	VU	0.58	0.16	0.09	Physeter macrocephalus	VU	0.73	0.56	0.41
Mesoplodon ginkgodens	M.gink	DD	0.54	0.16	0.09	Balaenoptera borealis	EN	0.71	0.57	0.40
Tursiops aduncus	T.adun	DD	0.73	0.12	0.09	Lagenorhynchus acutus	LC	0.63	0.62	0.39
Monodon monoceros	M.mono	LC	0.88	0.10	0.09	Mesoplodon europaeus	DD	0.50	0.78	0.39
Dugong dugon	D.dugo	VU	0.88	0.10	0.09	Orcinus orca	DD	0.67	0.56	0.37
Stenella coeruleoalba	S.coer	LC	0.50	0.17	0.08	Feresa attenuata	DD	0.50	0.74	0.37
Feresa attenuata	F.atte	DD	0.50	0.17	0.08	Balaenoptera edeni	DD	0.50	0.74	0.37
Balaenoptera edeni	B.eden	DD	0.50	0.16	0.08	Arctocephalus pusillus	LC	0.67	0.54	0.36
Tursiops truncatus	T.trun	LC	0.48	0.17	0.08	Stenella coeruleoalba	LC	0.50	0.72	0.36
Ziphius cavirostris	Z.cavi	LC	0.42	0.18	0.08	Pusa hispida	LC	0.83	0.43	0.35
Mesoplodon densirostris	M.dens	DD	0.46	0.17	0.08	Tursiops truncatus	LC	0.48	0.71	0.34
Globicephala melas	G.mela	DD	0.75	0.10	0.07	Pontoporia blainvillei	VU	0.67	0.50	0.34
Arctocephalus pusillus	A.pusi	LC	0.67	0.10	0.07	Monodon monoceros	LC	0.88	0.38	0.34
Sousa chinensis	S.chin	VU	0.60	0.11	0.07	Mesoplodon densirostris	DD	0.46	0.72	0.33
Kogia breviceps	K.brev	DD	0.38	0.17	0.06	Megaptera novaeangliae	LC	0.58	0.56	0.33
Kogia sima	K.sima	DD	0.38	0.16	0.06	Ursus maritimus	VU	0.83	0.39	0.32
Balaenoptera acutorostrata	B.acut	LC	0.35	0.17	0.06	Ziphius cavirostris	LC	0.42	0.69	0.29
Orcaella brevirostris	O.brev	EN	0.65	0.09	0.06	Kogia sima	DD	0.38	0.72	0.27
Arctocephalus tropicalis	A.trop	LC	0.63	0.09	0.05	Kogia breviceps	DD	0.38	0.72	0.27
Pontoporia blainvillei	P.blai	VU	0.67	0.08	0.05	Lagenorhynchus obscurus	DD	0.67	0.39	0.26
Tasmacetus shepherdi	T.shep	DD	0.81	0.05	0.04	Globicephala melas	DD	0.75	0.33	0.25
Eubalaena australis	E.aust	LC	0.79	0.05	0.04	Tasmacetus shepherdi	DD	0.81	0.29	0.23
Berardius arnuxii	B.arnu	DD	0.83	0.04	0.04	Otaria flavescens	LC	0.67	0.34	0.23
Hyperoodon planifrons	H.plan	LC	0.83	0.04	0.04	Arctocephalus tropicalis	LC	0.63	0.35	0.22
Arctocephalus gazella	A.gaze	LC	0.69	0.05	0.04	Caperea marginata	DD	0.75	0.29	0.22
Lobodon carcinophaga	L.carc	LC	0.92	0.04	0.04	Mesoplodon hectori	DD	0.63	0.33	0.21
Leptonychotes weddellii	L.wedd	LC	0.85	0.04	0.03	Balaenoptera bonaerensis	DD	0.69	0.30	0.21
Balaenoptera bonaerensis	B.bona	DD	0.69	0.05	0.03	Lontra felina	EN	0.56	0.37	0.21
Mirounga leonina	M.leon	LC	0.71	0.05	0.03	Arctocephalus australis	LC	0.67	0.31	0.20
Otaria flavescens	O.flav	LC	0.67	0.05	0.03	Phocoena spinipinnis	DD	0.50	0.41	0.20
Mesoplodon bowdoini	M.bowd	DD	0.63	0.05	0.03	Mesoplodon traversii	DD	0.63	0.32	0.20
Lagenorhynchus obscurus	L.obsc	DD	0.67	0.05	0.03	Balaenoptera acutorostrata	LC	0.35	0.56	0.20
Lagenorhynchus cruciger	L.cruc	LC	0.71	0.04	0.03	Arctocephalus forsteri	LC	0.63	0.29	0.18
Lissodelphis peronii	L.pero	DD	0.71	0.04	0.03	Lissodelphis peronii	DD	0.71	0.25	0.17
Ommatophoca rossii	O.ross	LC	0.81	0.04	0.03	Mesoplodon bowdoini	DD	0.63	0.28	0.17
Mesoplodon layardii	M.laya	DD	0.63	0.05	0.03	Eubalaena australis	LC	0.79	0.22	0.17
Phocoena dioptrica	P.diop	DD	0.63	0.05	0.03	Berardius arnuxii	DD	0.83	0.18	0.15
Lontra felina	L.feli	EN	0.56	0.05	0.03	Mesoplodon layardii	DD	0.63	0.23	0.15
Hydrurga leptonyx	H.lept	LC	0.83	0.03	0.03	Hyperoodon planifrons	LC	0.83	0.17	0.14
Caperea marginata	C.marg	DD	0.75	0.04	0.03	Mesoplodon grayi	DD	0.58	0.20	0.12
Phocoena spinipinnis	P.spin	DD	0.50	0.06	0.03	Lagenorhynchus cruciger	LC	0.71	0.15	0.11
Mesoplodon hectori	M.hect	DD	0.63	0.04	0.03	Phocoena dioptrica	DD	0.63	0.16	0.10
Mesoplodon grayi	M.gray	DD	0.58	0.05	0.03	Mirounga leonina	LC	0.71	0.12	0.09
Arctocephalus australis	A.aust	LC	0.67	0.04	0.02	Cephalorhynchus eutropia	NT	0.79	0.11	0.08
Orcaella heinsohni	O.hein	VU	0.58	0.02	0.01	Neophoca cinerea	EN	0.38	0.22	0.08
Arctocephalus forsteri	A.fors	LC	0.63	0.02	0.01	Lagenorhynchus australis	DD	0.75	0.10	0.07
Mesoplodon traversii	M.trav	DD	0.63	0.01	0.01	Arctocephalus philippii	LC	0.79	0.06	0.05
Cephalorhynchus hectori	C.hect	EN	0.75	0.01	0.01	Cephalorhynchus commersonii	LC	0.79	0.06	0.05
Cephalorhynchus eutropia	C.eutr	NT	0.79	0.00	0.00	Arctocephalus gazella	LC	0.69	0.05	0.04
Cephalorhynchus commersonii	C.comm	LC	0.79	0.00	0.00	Hydrurga leptonyx	LC	0.83	0.04	0.03
Neophoca cinerea	N.cine	EN	0.38	0.00	0.00	Phocarctos hookeri	EN	0.71	0.04	0.03
Arctocephalus philippii	A.phil	LC	0.79	0.00	0.00	Lobodon carcinophaga	LC	0.92	0.02	0.02
Lagenorhynchus australis	L.aust	DD	0.75	0.00	0.00	Leptonychotes weddellii	LC	0.85	0.02	0.02
Phocarctos hookeri	P.hook	EN	0.71	0.00	0.00	Ommatophoca rossii	LC	0.81	0.02	0.02

Fig. S5 | Vulnerability to global warming against evolutionary distinctivness and functional originality for marine mammals. The first line represents the marine mammals' vulnerability to global warming against the evolutionary distinctiveness for the end of the century (2070-2099) for the RCP2.6 (a) and for the RCP8.5 (b). The second line represent, for the end of the century (2070-2099), the marine mammals' vulnerability to global warming against the functional originality distinctiveness for the RCP2.6 (c) and for the RCP8.5 (d). The blue rectangle highlights the less vulnerable species. The yellow rectangles highlight either the most phylogenetically or functionally original species in x-axis or the most vulnerable species to global warming in y-axis. Hence, species in the red rectangle are highly vulnerable to global warming and highly phylogenetically or functionally original. See full names for the abbreviations in Appendix 5.

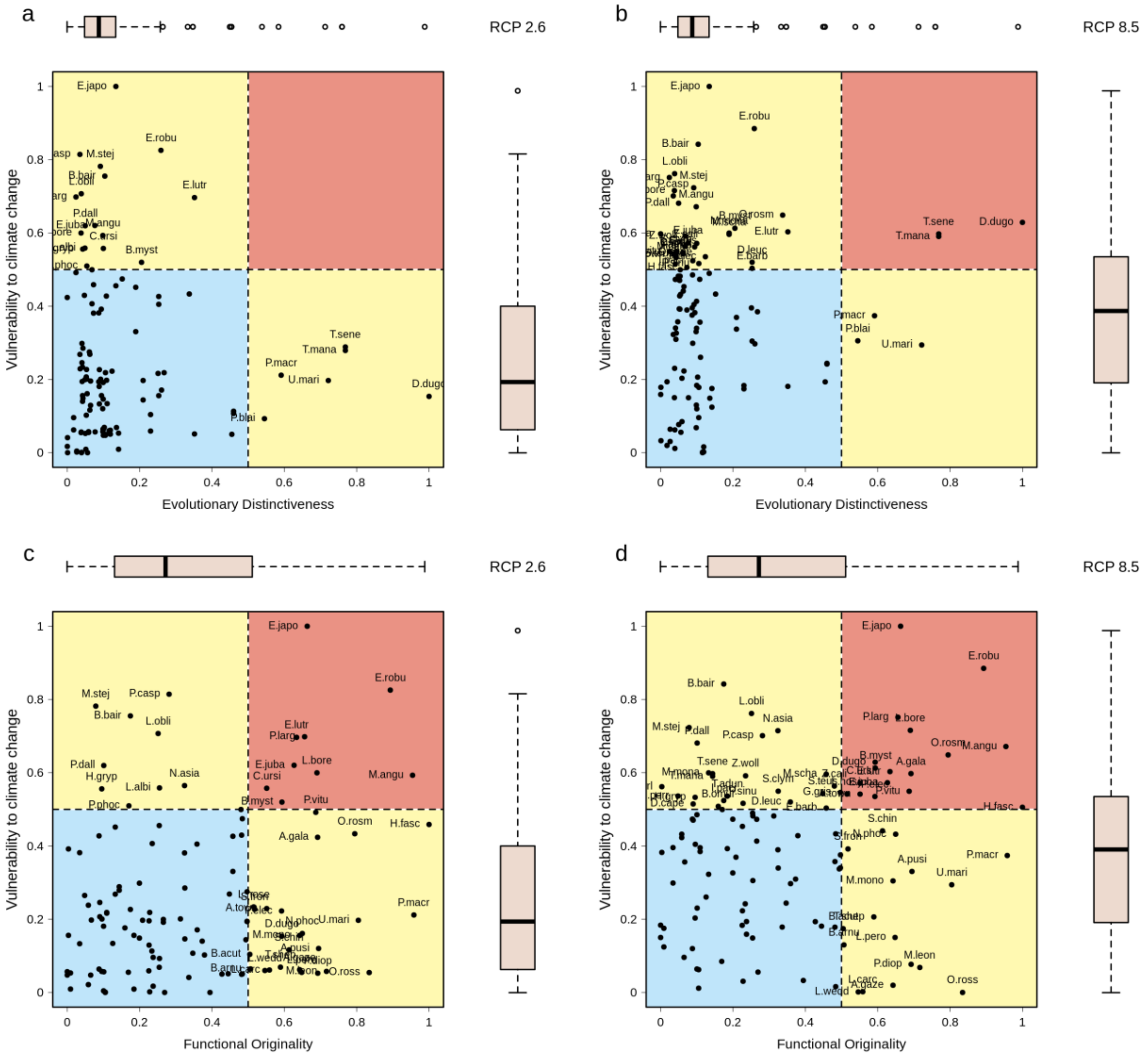

Fig. S6 | Scenario of potential loss of functional richness and phylogenetic diversity in the global marine mammal fauna

Potential loss of functional richness (A, B) and phylogenetic diversity (C, D) in the global marine mammal fauna according to the RCP2.6 scenario for the end of the century (2070-2099). The upper graphs (A, C) show two erosion scenarios, one repeated 999 times where order of species extinction was selected randomly (dark continuous line) with the corresponding standard deviation and a second where species extinction was selected according to their vulnerability to global warming (green continuous line). The lower graphs (B, D) show two random erosion scenarios. One repeated 999 times where order of species extinction was selected randomly (dark continuous line) and a second with species being grouped according to their IUCN category (Critically Endangered (CR), Endangered (EN), Vulnerable (VU), Near Threatened (NT), Least Concern (LC) and Data Deficient (DD)) prior to randomization. Finally, the lower graph present a scenario ("Vulnerability & IUCN", multicolor line) with species grouped by IUCN categories and decreasingly ordered within each group according to their vulnerability to global warming. Ranking of species differ between each pair of vulnerability scenarios, preventing from direct comparison.

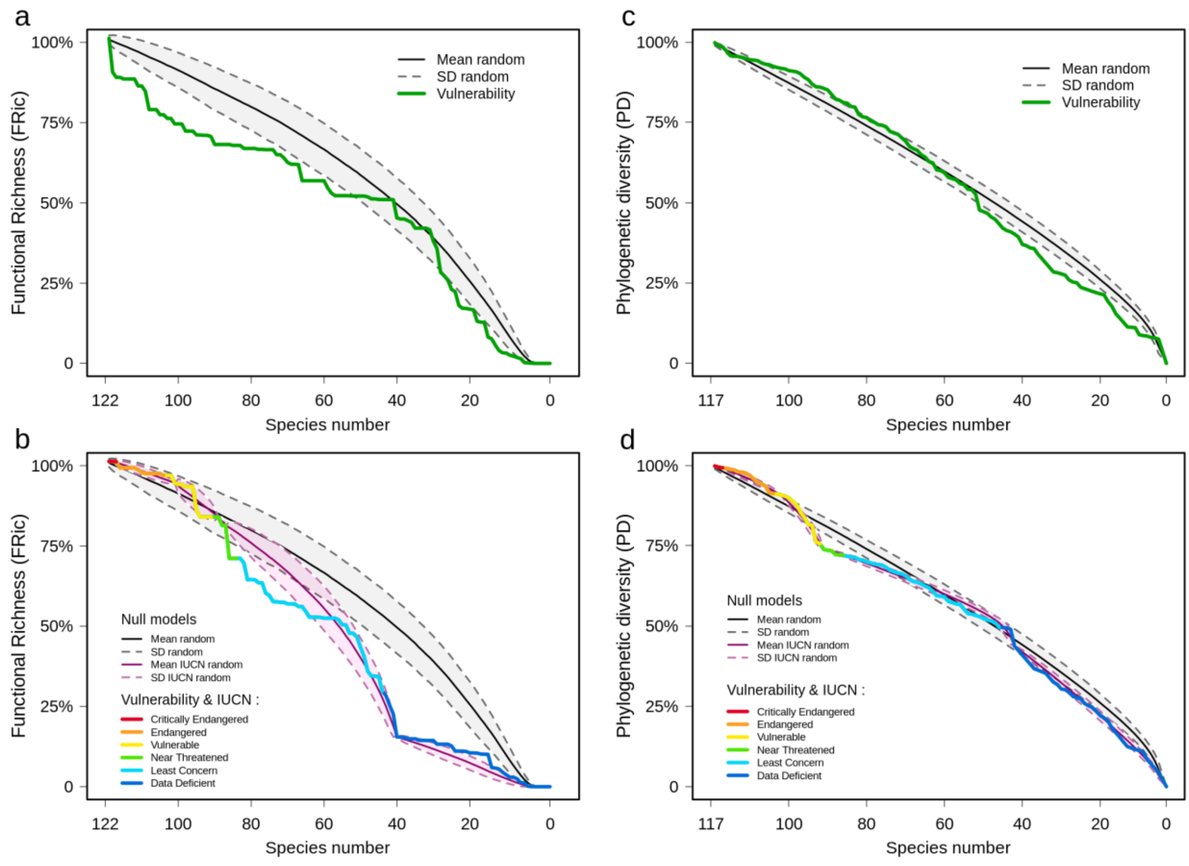

References Supplementary Information

1. Laidre, K. L. *et al.* Quantifying the sensitivity of Arctic marine mammals to climate-induced habitat change. *Ecol. Appl.* **18**, S97-125 (2008).
2. Pavés, H.J. & González, H.E. Carbon fluxes within the pelagic food web in the coastal area off Antofagasta (23°S), Chile: The significance of the microbial versus classical food webs. *Ecol. Modell.* **212**, 218–232 (2008).
3. Chessman, B.C. Identifying species at risk from climate change: Traits predict the drought vulnerability of freshwater fishes. *Biol. Conserv*. **160**, 40–49 (2013).
4. Olden, J.D., Poff, N.L. & Bestgen, K.R. Trait synergisms and the rarity, extirpation, and extinction risk of desert fishes. *Ecology* **89**, 847–56 (2008).
5. Pauly, D. *et al*. Diet composition and trophic levels of marine mammals. *ICES J. Mar. Sci*. **55**, 467–481 (1998).
6. Rosset, V. & Oertli, B. Freshwater biodiversity under climate warming pressure: Identifying the winners and losers in temperate standing waterbodies. *Biol. Conserv.* **144**, 2311–2319 (2011).
7. Peters, R.L. The Greenhouse Effect and Nature Reserves. *Bioscience* **35**, 707–717 (1985).
8. Garcia, R.A., Araújo, M.B., Burgess, N.D., Foden, W.B., Gutsche, A., Rahbek, C., and Cabeza, M. Matching species traits to projected threats and opportunities from climate change. *J. Biogeogr*. **41**, 724–735 (2014).
9. Davidson, A.D. *et al*. Drivers and hotspots of extinction risk in marine mammals. *Proc. Natl. Acad. Sci*. **109**, 3395–3400 (2012).
10. Schumann, N. *et al.* Impacts of climate change on Australian marine mammals. *Aust. J. Zool*. **61**, 146 (2013).
11. Kaschner, K.B *et al*. AquaMaps Environmental Dataset: Half-Degree Cells Authority File (HCAF). ver. 5 World Wide Web Electron. Publ., Version 7, (2013).
12. Schumann, N. *et al.* Impacts of climate change on Australian marine mammals. *Aust. J. Zool.* **61**, 146 (2013).
13. IPCC. Fifth Assessment Report Climate Change 2013 : the Physical Science Basis.(2013)
14. Cheung, W. *et al*. Intrinsic vulnerability in the global fish catch. *Mar. Ecol. Prog. Ser.* **333**, 1–12 (2007).
15. Simmonds, M.P. & Isaac, S.J. The impacts of climate change on marine mammals: early signs of significant problems. *Oryx* **41**, 19 (2007)
16. Burek, K.A., Gulland, F.M.D. & O'Hara, T.M. Effects of climate change on arctic marine mammal health. *Ecol. Appl*. **18**, S126–S134 (2008).

17. González-Suárez, M., Gómez, A. & Revilla, E. Which intrinsic traits predict vulnerability to extinction depends on the actual threatening processes. *Ecosphere* **4**, art76 (2013)

18. Warren, M.S. *et al.* Rapid responses of British butterflies to opposing forces of climate and habitat change. *Nature* **414**, 65–69 (2001).

19. Opdam, P. & Wascher, D. Climate change meets habitat fragmentation: linking landscape and biogeographical scale levels in research and conservation. *Biol. Conserv.* **117**, 285–297 (2004).

20. Ribic, C.A., Ainley, D.G. & Fraser, W.R. Habitat selection by marine mammals in the marginal ice zone. *Antarct. Sci. **3**,* 181–186 (1991).

21. Murase, H., Matsuoka, K., Ichii, T. & Nishiwaki, S. Relationship between the distribution of euphausiids and baleen whales in the Antarctic (35°E – 145°W). P*olar Biol*. **25**, 135–145 (2002).

22. Kaschner, K. *et al.* Current and Future Patterns of Global Marine Mammal Biodiversity. *PLoS One* **6**, e19653 (2011).

23. Perrin, W.F., Würsig, B.G. & Thewissen, J.G.M. Encyclopedia of marine mammals (Elsevier/Academic Press) (2009).

24. Hutchings, J.A. *et al*. Life-history correlates of extinction risk and recovery potential. *Ecol. Appl.* **22**, 1061–1067 (2012).

25. Vasquez, R., and Simonetti, J.A. (1999). Life history traits and sensitivity to landscape change: the case of birds and mammals of mediterranean. *Chile. Rev. Chil. Hist. Nat.* **72**, 517–525.

26. Davidson, A.D. *et al*. Drivers and hotspots of extinction risk in marine mammals. *Proc. Natl. Acad. Sci. U. S. A.* **109**, 3395–400 (2012).

27. Cheung, W.W.L., Pauly, D. & Sarmiento, J.L. (2013). How to make progress in projecting climate change impacts. ICES J. *Mar. Sci. J. du Cons*. **70**, 1069–1074.
